# Analysis of Network Topologies Underlying Ethylene Growth Response Kinetics

**DOI:** 10.3389/fpls.2016.01308

**Published:** 2016-08-30

**Authors:** Aaron M. Prescott, Forest W. McCollough, Bryan L. Eldreth, Brad M. Binder, Steven M. Abel

**Affiliations:** ^1^Department of Chemical and Biomolecular Engineering, University of TennesseeKnoxville, TN, USA; ^2^Department of Biochemistry and Cellular and Molecular Biology, University of TennesseeKnoxville, TN, USA; ^3^National Institute for Mathematical and Biological Synthesis, University of TennesseeKnoxville, TN, USA

**Keywords:** ethylene, signal transduction, network topologies, computational modeling, evolutionary algorithm

## Abstract

Most models for ethylene signaling involve a linear pathway. However, measurements of seedling growth kinetics when ethylene is applied and removed have resulted in more complex network models that include coherent feedforward, negative feedback, and positive feedback motifs. The dynamical responses of the proposed networks have not been explored in a quantitative manner. Here, we explore (i) whether any of the proposed models are capable of producing growth-response behaviors consistent with experimental observations and (ii) what mechanistic roles various parts of the network topologies play in ethylene signaling. To address this, we used computational methods to explore two general network topologies: The first contains a coherent feedforward loop that inhibits growth and a negative feedback from growth onto itself (CFF/NFB). In the second, ethylene promotes the cleavage of EIN2, with the product of the cleavage inhibiting growth and promoting the production of EIN2 through a positive feedback loop (PFB). Since few network parameters for ethylene signaling are known in detail, we used an evolutionary algorithm to explore sets of parameters that produce behaviors similar to experimental growth response kinetics of both wildtype and mutant seedlings. We generated a library of parameter sets by independently running the evolutionary algorithm many times. Both network topologies produce behavior consistent with experimental observations, and analysis of the parameter sets allows us to identify important network interactions and parameter constraints. We additionally screened these parameter sets for growth recovery in the presence of sub-saturating ethylene doses, which is an experimentally-observed property that emerges in some of the evolved parameter sets. Finally, we probed simplified networks maintaining key features of the CFF/NFB and PFB topologies. From this, we verified observations drawn from the larger networks about mechanisms underlying ethylene signaling. Analysis of each network topology results in predictions about changes that occur in network components that can be experimentally tested to give insights into which, if either, network underlies ethylene responses.

## 1. Introduction

**Figure 1:**
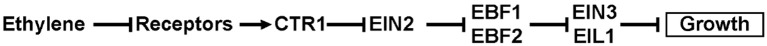
**Basic linear ethylene signaling network**.

Ethylene is the simplest of olefin gases and functions as a plant hormone, affecting many processes throughout the lifetime of a plant including seed germination, growth, formation of the apical hook, senescence, fruit ripening, abscission, and responses to various stresses (Mattoo and Suttle, 1991; Abeles et al., 1992). Ethylene inhibits the growth of dark-grown eudicot seedlings (Abeles et al., 1992), and sustained exposure to ethylene leads to a growth-inhibition response that has been used to screen for mutants and to provide information about the ethylene signaling network (Bleecker et al., 1988; Guzman and Ecker, 1990). Most proposed models of ethylene signaling consist of a linear pathway (Figure 1), where in air, ethylene receptors signal to the CONSTITUTIVE RESPONSE1 (CTR1) protein kinase which functions as a negative regulator of ethylene signaling (Kieber et al., 1993). CTR1 prevents ethylene signaling by phosphorylating the ETHYLENE INSENSITIVE2 (EIN2) protein, leading to its ubiquitination and proteolysis (Chen et al., 2011; Ju et al., 2012; Qiao et al., 2012). The binding of ethylene to ethylene receptors reduces the activity of the receptors, leading to reduced activity of CTR1 kinase and reduced phosphorylation of EIN2 protein (Chen et al., 2011; Ju et al., 2012; Qiao et al., 2012). The reduction in EIN2 phosphorylation leads to a decrease in ubiquitination of EIN2, causing a rise in EIN2 protein levels and allowing for proteolytic release of the C-terminal portion (EIN2-C) of the protein (Qiao et al., 2009; Ju et al., 2012; Qiao et al., 2012; Wen et al., 2012). EIN2-C affects the levels of two transcription factors, EIN3 and EIN3-Like1 (EIL1), in part by regulating their ubiquitination via S-PHASE KINASE-ASSOCIATED1-CULLIN-F-BOX (SCF) ubiquitin ligase complexes containing EIN3-BINDING F-BOX1 and 2 (EBF1 and 2) F-box proteins (Guo and Ecker, 2003; Potuschak et al., 2003; Yanagisawa et al., 2003; Gagne et al., 2004; Binder et al., 2007; An et al., 2010). In the presence of ethylene, ubiquitination of EIN3 and EIL1 is reduced, leading to accumulation of these transcription factors causing most ethylene responses (Guo and Ecker, 2003; Potuschak et al., 2003; Yanagisawa et al., 2003; Binder et al., 2004a, 2007; Gagne et al., 2004; An et al., 2010).

The above model was developed based on end-point analyses. Even though end-point analysis of ethylene responses continues to be an instructive bioassay, it is limited because transient events are overlooked. Time-lapse imaging has provided information about the kinetics of ethylene growth responses. The kinetics of ethylene responses have been studied for several plant species (Laan, 1934; Warner and Leopold, 1971; Burg, 1973; Goeschl and Kays, 1975; Rauser and Horton, 1975; Jackson, 1983) and most extensively studied in the model flowering plant, *Arabidopsis thaliana* (Binder et al., 2004a,b; Potuschak et al., 2006; Binder et al., 2007; Gao et al., 2008; Christians et al., 2009; Vandenbussche et al., 2010; van Zanten et al., 2010; Žádníková et al., 2010; Kim et al., 2011, 2012; McDaniel and Binder, 2012; Bakshi et al., 2015; Merchante et al., 2015; Rai et al., 2015).

**Figure 2:**
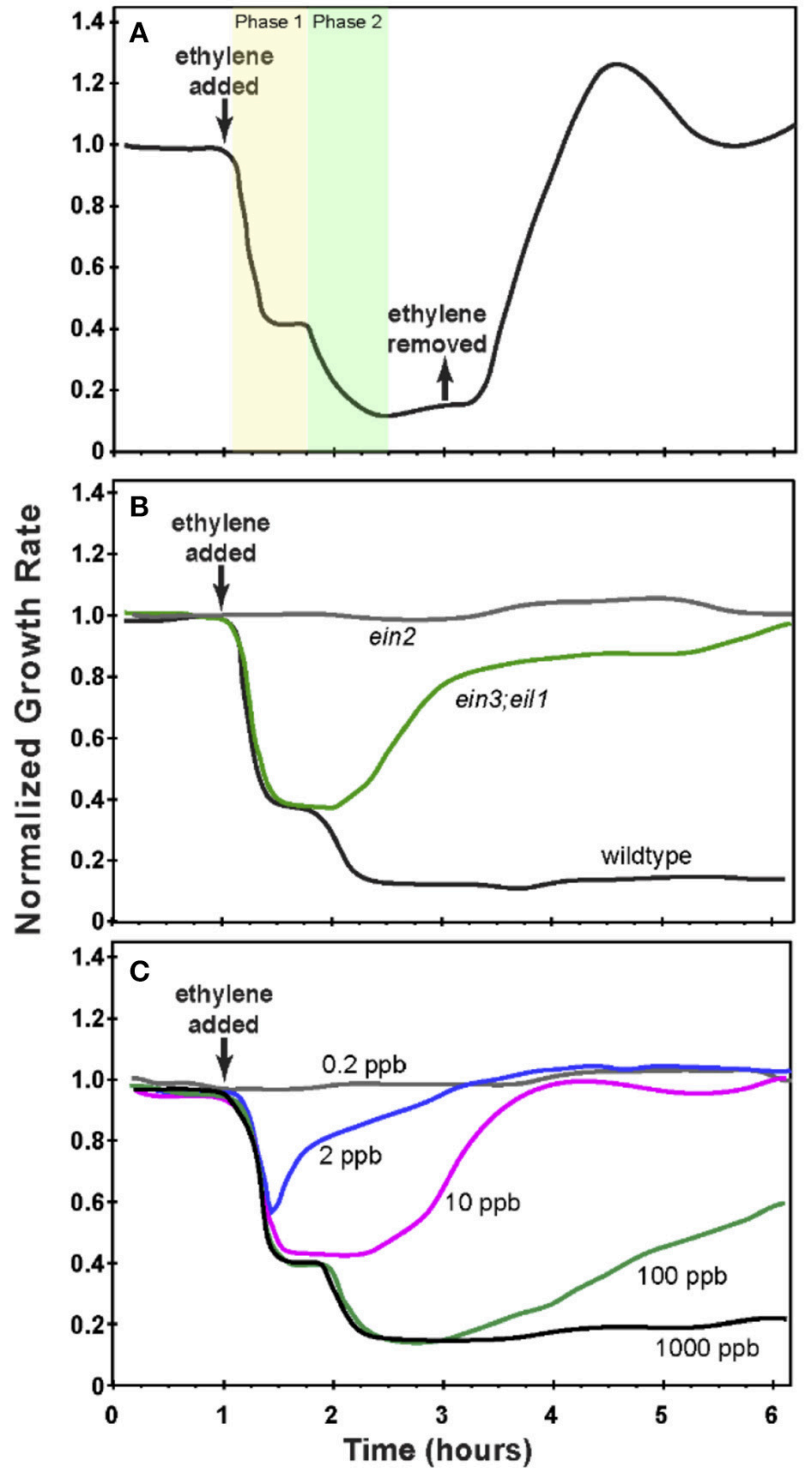
**Growth response kinetics of dark-grown ***Arabidopsis*** seedling hypocotyls. (A)** The normalized growth rate of wildtype Arabidopsis. Seedlings were grown in air for 1 h at which time 10 ppm ethylene was applied for 2 h at which time ethylene-free air was used to replace the ethylene. **(B)** The normalized growth rate of wildtype *Arabidopsis* compared to *ein3;eil1* and *ein2* mutants. Seedlings were grown in air for 1 h at which time 10 ppm ethylene was applied. **(C)** The normalized growth rate of wildtype Arabidopsis treated with varying concentrations of ethylene as indicated. Seedlings were grown in air for 1 h prior to application of ethylene. In all panels, the growth rate was normalized to the growth rate in air prior to treatment with ethylene. Based on data from Binder et al. (2004a,b).

Studies of ethylene growth kinetics in Arabidopsis have revealed two phases of growth inhibition at saturating ethylene levels (1 ppm or above, see Figure 2A) (Binder et al., 2004b). The first phase is rapid, with a decrease in growth rate beginning approximately 10 min after the application of ethylene and lasting approximately 10 min, at which point the growth rate reaches a plateau. This plateau lasts approximately 30 min when a second, slower phase of growth inhibition is observed. Approximately 90 min after the application of ethylene, the growth rate reaches a minimum that lasts for as long as saturating levels of ethylene are present. If ethylene is removed after 2 h, seedlings recover to pre-treatment growth rates in approximately 90 min (Figure 2A).

The two phases of growth inhibition are genetically separable (Binder et al., 2004a). Mutants lacking EIN3 and EIL1 (*ein3;eil1*) have a normal first phase of growth inhibition but fail to have a second phase response and over time return to pre-treatment growth rates in the continued presence of ethylene (Figure 2B). This demonstrates that the first phase of growth inhibition is EIN3/EIL1-independent. Mutants lacking EIN2 (*ein2*) have no response to ethylene. Additionally, in wild type plants, adaptation is observed at sub-saturating levels of ethylene. At intermediate to high sub-saturating levels (e.g., 100 ppb), seedlings initially show both phases of growth inhibition but then have a partial recovery to an intermediate growth rate. At lower levels of ethylene (e.g., 2 and 10 ppb), only the first phase of growth inhibition occurs and is followed by recovery of the growth rate (Figure 2C) (Binder et al., 2004a).

The experiments described above and other observations that indicate possible alternative pathways and feedback control (Kieber et al., 1993; Roman et al., 1995; Larsen and Chang, 2001; Hall and Bleecker, 2003; Qiu et al., 2012; Rai et al., 2015) suggest that ethylene signal transduction is not simply a linear pathway. Several network models have been proposed that involve more complicated topologies (Binder et al., 2004a; Gao et al., 2008; Kim et al., 2012). These and related networks are the focus of this paper.

**Figure 3:**
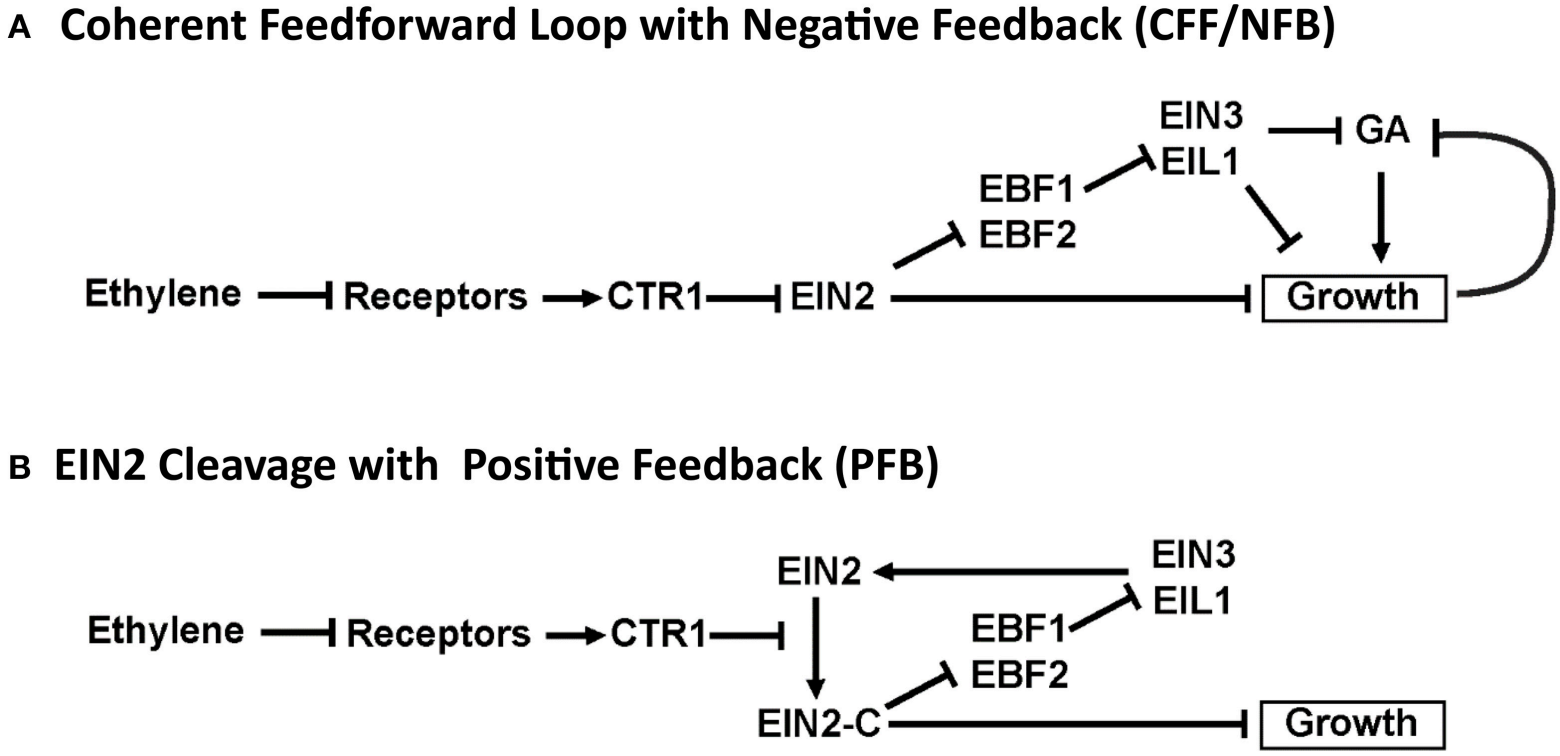
**Diagrams illustrating proposed network topologies**.

A study comparing the time-dependent growth responses of several plant species led to a proposed network that included both coherent feedforward and negative feedback (CFF/NFB) signaling motifs (Figure 3A; Kim et al., 2012). In the CFF/NFB model, in air (i.e., without ethylene) the receptors signal to CTR1, which in turn inhibits downstream signaling. This leads to fast growth with feedback on growth occurring via the modulation of gibberellin (GA), a hormone known to stimulate growth. Application of ethylene inhibits the receptors, leading to a reduction of CTR1 activity and hence an increase in EIN2 levels. EIN2 is predicted to cause an initial growth inhibition response independently of EIN3 and EIL1. EIN2 also inhibits the EBF1 and EBF2 F-box proteins, leading to increases in EIN3 and EIL1. In the CFF/NFB network model, EIN3 and EIL1 inhibit growth via a GA-dependent and GA-independent pathway. The indirect inhibition of growth from EIN2 through EIN3 and EIL1 is hypothesized to be responsible for the second phase of growth inhibition. Qualitatively, this network topology provides a framework to understand the molecular basis for phase 1 and phase 2 growth inhibition and its regulation by the coherent feedforward signal. It also provides a mechanism for transient growth inhibition in the absence of EIN3 and EIL1 that is regulated by negative feedback components.

We were curious to determine whether other network topologies would yield response kinetics similar to experiments. We previously proposed a model where one function of EIN3 and EIL1 is to provide feedback to regulate growth (Binder et al., 2004a). More recent data has shown that ethylene signaling requires the accumulation of EIN2 followed by the proteolytic cleavage of the EIN2 C-terminal tail (Alonso et al., 1999; Ju et al., 2012; Qiao et al., 2012; Wen et al., 2012). This accumulation of EIN2 protein is reduced in *ein3;eil1* double mutants (Qiao et al., 2009) suggesting a mechanism for feedback by EIN3 and EIL1. We therefore developed a network topology where the function of EIN3 and EIL1 is to provide feedback stimulation in the form of increased synthesis of EIN2 (Figure 3B). EIN2-C feeds back through EBF1/2 and EIN3 to promote EIN2. Without accumulation of EIN2, the prediction is that the levels of EIN2-C would decrease, resulting in growth reversal. We refer to this network as the cleavage with positive feedback (PFB) network.

Few computational models of ethylene signaling have been published (Díaz and Álvarez-Buylla, 2006; Díaz and Alvarez-Buylla, 2009), and none take into account the dynamical information obtained from kinetic studies of ethylene response. Considering experimental results of this nature provides an opportunity to identify network features underlying the observed growth responses. Therefore, we developed computational models that account for proposed interactions within the CFF/NFB and PFB networks described above. These networks are modeled as sets of coupled ordinary differential equations (ODEs) describing the time evolution of network components. We are interested in (i) whether the proposed networks are capable of producing dynamical growth-response behavior consistent with experiments, and (ii) mechanisms underlying the network response when it does recapitulate experimental results. The CFF/NFB and PFB networks we consider have relatively high-dimensional parameter spaces (35 and 26 parameters, respectively). The parameters regulate numerous coupled, nonlinear ODEs describing the dynamics of the network, and changing the value of one parameter can have unexpected effects on network responses. Few *in vivo* measurements of network parameters are available. As such, we used an evolutionary algorithm (EA) to search for sets of parameters that produce network behavior consistent with experimental growth response kinetics. EAs are a class of numerical optimization techniques and have been used to investigate networks in a variety of biochemical applications (Bäck and Schwefel, 1993; Bray and Lay, 1994; François and Hakim, 2004; Patil et al., 2005; Auliac et al., 2008; Sun et al., 2012; Spirov and Holloway, 2013; Feng et al., 2015). For example, time-course data has been used to determine parameters of small genetic networks (Kikuchi et al., 2003) and parameters associated with signal transduction in neurons (Arisi et al., 2006).

We used an EA to evolve parameter sets that produce ethylene growth responses similar to those observed in experiments. In particular, we focused on evolving two-phase growth inhibition (2-PGI) and *ein3;eil1* mutant partial growth recovery (MPGR). By repeatedly performing independent runs of our EA, we created libraries of evolved parameter sets. We gain insight into mechanisms underlying network responses by analyzing the dynamics of individual network components and the distributions of parameters governing the network. We additionally screen the parameter sets for partial growth recovery in the presence of sub-saturating ethylene doses, which is a property that emerges in some of the evolved parameter sets. We further explore each network by identifying simplified networks producing both 2-PGI and MPGR.

## 2. Methods

### 2.1. Kinetics of the ethylene response network

We model the ethylene growth-response networks proposed above using systems of coupled ordinary differential equations (ODEs). Ethylene concentration is treated as an input variable that is varied to mimic experimental conditions. We treat the growth rate, denoted by [Growth], as a concentration-like variable that measures the fraction of maximal growth rate. The unbound ethylene receptor concentration is described by a production term and a mass-action binding term representing ethylene binding. The time-dependence of all other components is described by production and degradation terms. As an example, the ODE describing CTR1 dynamics is written

$$\begin{array}{l} \frac{d[\text{CTR1}]}{dt}=\;{\left(k_{\text{prod}}\frac{{\left[\text{R}\right]}^{N}}{K_{\text{prod}}^{N}+{\left[\text{R}\right]}^{N}}\right)}_{\text{R}}(1-[\text{CTR1}])\\ \;-k_{\text{degr}}^{\text{CTR1}}[\text{CTR1}] \end{array}$$

Concentrations are denoted by square brackets, *k* denotes a reaction rate, *K* denotes an activation coefficient in a Hill equation, and *N* is the associated Hill coefficient. All concentrations are restricted to the range of 0 to 1 and can be interpreted as the fraction of the maximum concentration for each species. The concentration range is constrained by describing the production as a logistic production term, with production vanishing when the concentration approaches 1. Interactions between network components are described with Hill-like kinetics. For example, in the above equation, CTR1 is promoted by receptors (R), which is captured by the Hill equation in the production term on the right-hand side. Inhibitory interactions promote the rate of degradation (e.g., EBF increases the degradation rate of EIN3 and EIL1). Stimulatory interactions promote the rate of production. The complete sets of ODEs for the networks studied are included in the Supplementary Material. We initially equilibrate the system with no ethylene present, allowing it to reach steady state. We then introduce a step-change in ethylene to mimic experimental conditions. We treat EIN3 and EIL1 as a single entity, and therefore, to model the *ein3;eil1* mutant, the production rate for EIN3 is set equal to zero.

### 2.2. Evolutionary algorithm

Given the system of ODEs describing network dynamics, we use an evolutionary algorithm (EA) to identify sets of parameters that produce growth-response behavior similar to that observed experimentally. EAs are a class of optimization techniques that utilize the principle of inherited fitness to optimize parameters. A population of parameter sets is evolved over multiple generations. At each generation, each parameter set is evaluated by a fitness function and ranked by its fitness. Parameter sets with better rankings are modified in order to produce a new population of parameter sets for evaluation. The modifications consist of mutation and crossover operations. Mutations change a parameter value within a set to a new, randomly sampled value. Crossovers are events in which subsets from two high-performing parameter sets are recombined to form a new parameter set. Details of the mutations and crossovers depend on the specific implementation of the EA. Each iteration in which the population of parameter sets is updated is termed a generation.

We used the following fitness function for all proposed ethylene signaling networks:

$$\begin{array}{l} \text{fitness}=\sum_{i=1}^{N_{\text{wt}}}\alpha_{i}{\left({\left[\text{Growth}\right]}_{\text{calc}}(t_{i})-{\left[\text{Growth}\right]}_{\text{targ}}(t_{i})\right)}_{\text{wt}}^{2}\\ \;+\sum_{i=1}^{N_{\text{mt}}}\beta_{i}{\left({\left[\text{Growth}\right]}_{\text{calc}}(t_{i})-{\left[\text{Growth}\right]}_{\text{targ}}(t_{i})\right)}_{\text{mt}}^{2} \end{array}$$

Calculated growth values ([*Growth*]_calc_(*t*_*i*_)]) are determined by numerically solving the system of ODEs in MATLAB using the ode45 numerical solver. Target values ([*Growth*]_targ_(*t*_*i*_)]) were obtained using experimentally-determined growth rates at select times (Figures 2A,B). Target values were selected from wildtype (wt) and *ein3;eil1* mutant (mt) experiments, with the index *i* in each sum indexing the target values (there are *N*_wt_ target values for the wildtype response and *N*_mt_ target values for the mutant response). For each time point, the squared deviation of the calculated growth rate from the target value is multiplied by a weighting factor (α_*i*_, β_*i*_) that emphasizes important regimes of the growth response. Specifically, we emphasize pre-ethylene steady state values, the wildtype two-phase growth inhibition response, minimal growth rate following ethylene introduction, and maximum growth recovery levels. Target values and weighting factors are provided in Supplemental Tables 2.1.1, 2.1.2. Target growth rates were scaled by dividing all experimental growth rates by the maximum observed growth rate under both wildtype and mutant conditions. This gives a pre-ethylene growth rate target value that is less than one, in contrast with Figure 2 in which growth rates were scaled so that pre-ethylene growth rates were unity. The evolutionary algorithm was designed to minimize the fitness function.

In our EA, we use a population size of 200 parameters sets at each generation. The initial parameter sets are generated by selecting uniformly distributed random values for each parameter from allowed parameter ranges (see Supplemental Table 2.1.3). Hill coefficients (N) are restricted to integer values. After evaluating each parameter set, the 50 parameter sets with the lowest fitness scores are selected as source parameter sets. These source sets are used to generate the population of parameter sets for the next generation. New parameter sets are produced by performing a two-point crossover followed by mutations. The crossover events and mutations allow a balance of global and local exploration of parameter space, and the algorithm converged to local minima of the fitness function for the signaling networks studied. Two-point crossovers are performed by randomly selecting two source parameter sets with replacement (i.e., the same set can be chosen twice). Two crossover points are chosen at random from the list of parameters. Two blocks of parameters are taken from the first source and the other block is taken from the second source, leading to a newly constructed set of parameters. Additionally, the probability of mutation for each parameter is chosen such that on average three parameters within the crossover product are mutated (the probability is 3/35 for the CFF/NFB network and 3/26 for the PFB network). When a parameter is selected for mutation, the decision to increase or decrease the value is made with equal probability. The parameter is then multiplied or divided, respectively, by a uniformly distributed value between 1 and 2. When this decision would result in a parameter exceeding its upper bound, a uniform random value between the current parameter value and its upper limit is used instead. The source parameter sets are updated each generation by replacing 25 randomly selected source sets with parameter sets having the lowest fitness scores from the current population.

We ran the EA for 400 generations and recorded the parameter set with the lowest fitness score for additional analysis. This evolutionary process was repeated independently 500–4000 times depending on the network topology. Each parameter set was screened for the targeted network behavior and the resulting data was used to characterize ethylene response kinetics and identify features of the evolved parameters.

### 2.3. Screening for targeted responses

After running the EA, we check whether the resulting growth responses exhibit wildtype two-phase growth inhibition and/or *ein3;eil1* mutant partial growth recovery. Specifically, network responses are checked to ensure that the wildtype response meets the following conditions:

1. With no ethylene, steady state growth is sufficiently high.
2. After applying ethylene, the minimal growth rate is sufficiently low.
3. A plateau-like region separates the first and second phases of growth inhibition.
4. Following removal of ethylene, growth recovers to a sufficiently high level.

Logic diagrams for discriminant functions are included in Supplemental Figures S1, S2. For mutant behavior, traits 1 and 2 were used to check for proper behavior. The discriminant functions were designed to make the inclusion of false positives unlikely.

### 2.4. Ethylene dose response

We additionally screen evolved parameters sets to identify whether they exhibit sub-saturating ethylene dose response kinetics similar to experimental observations (see Figure 2C). The level of ethylene that leads to a sub-saturating response depends on the parameters of the network. Thus, we first identify the range of ethylene concentrations over which the network is responsive to concentration variations. For each evolved parameter set, we use a binary search method to identify the ethylene concentration range in which (i) the maximum dose produces long-time growth rate between 0.5 and 1.0% above minimum growth observed in the saturated response and (ii) the minimum dose produces a minimum growth rate between 0.5 and 1.0% below pre-ethylene steady state growth. We consider 20 evenly distributed ethylene concentrations between these bounds to test for partial growth recovery in the presence of sustained ethylene exposure. A parameter set is considered to exhibit sub-saturating growth recovery if there exists at least one ethylene concentration at which the growth maximum that occurs 1 h or longer after the introduction of ethylene exceeds the minimum growth observed within the first hour of ethylene exposure by at least 0.1 (maximum possible growth is unity).

## 3. Results

### 3.1. Coherent feedforward/negative feedback network

The CFF/NFB network (Figure 3A) was proposed by Kim et al. (2012) based on growth kinetics in response to the addition and removal of ethylene. It can be broken down into three distinct regions: (i) the initial linear signaling cascade consisting of ethylene receptors, CTR1, and EIN2; (ii) a coherent feedforward loop with EIN2 as the initial node that inhibits growth both directly and indirectly (via EBF and EIN3); (iii) a negative feedback loop consisting of growth and GA. The coherent feedforward cascade interacts with the negative feedback loop as a result of the inhibitory effect of EIN3 on GA. As indicated in Figure 3A, we treat EBF1 and EBF2 as well as EIN3 and EIL1 as single entities. We refer to these nodes as EBF and EIN3, respectively. This reduces the complexity of the model and the dimensionality of the parameter space while keeping key topological features of the network. Additionally, with existing experimental data, it is difficult to elucidate differences between these individual components, which could be included in a more detailed computational model.

**Figure 4:**
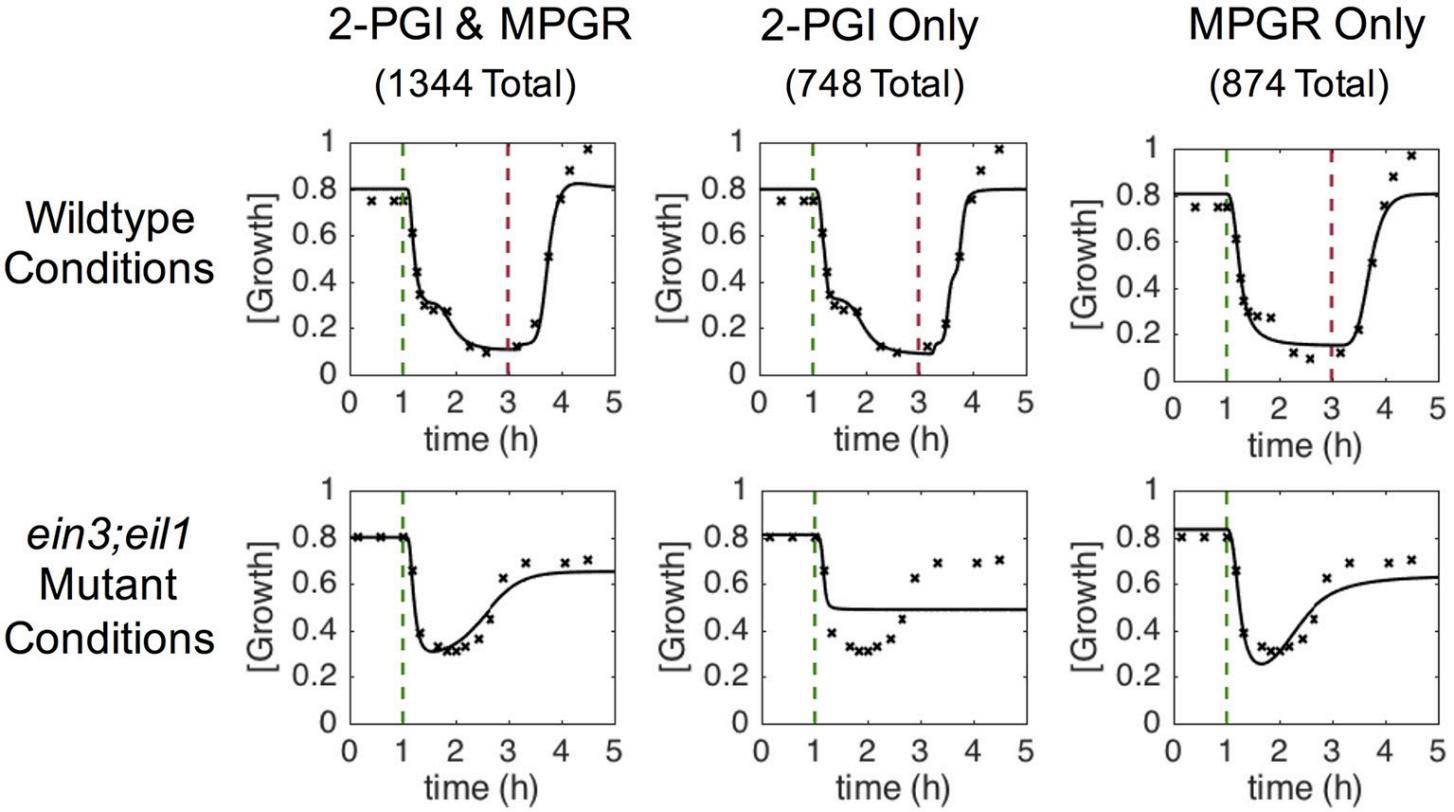
**Characteristic growth responses at saturating ethylene doses**. Columns show examples of time-dependent growth responses passing different combinations of screening criteria. Each column corresponds to a single set of evolved parameters. Rows show different simulated conditions (wildtype and *ein3;eil1* mutant). Targeted growth rates are denoted by x and the evolved response is shown by solid lines. Dashed vertical lines indicate time points at which ethylene was introduced (green) and removed (red).

We conducted multiple independent trials of the EA, obtaining 3774 sets of optimized parameters. Using the discriminant functions described previously, each evolved parameter set was screened for wildtype two-phase growth inhibition (2-PGI) and *ein3;eil1* mutant partial growth recovery (MPGR). Figure 4 shows examples of results that exhibit both 2-PGI and MPGR, as well as those that exhibit only one of the responses. Approximately 36% of the evolved parameter sets exhibit both 2-PGI and MPGR, 20% exhibit only 2-PGI, and 23% exhibit only MPGR. The large number of parameter sets yielding one or both of the targeted growth responses provides a large data set for analysis.

#### 3.1.1. Dynamical response of network components

**Figure 5:**
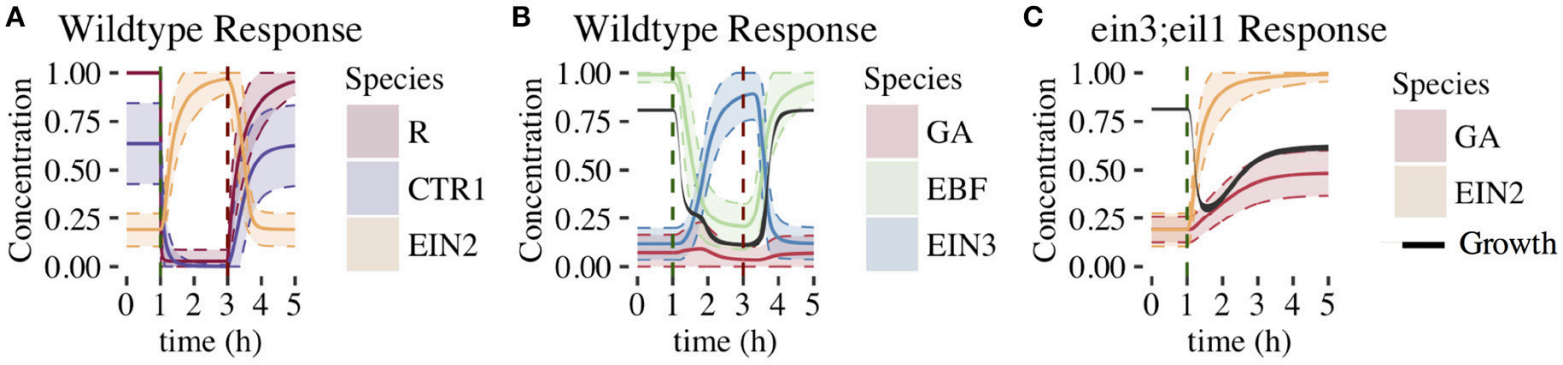
**Time evolution of CFF/NFB network components**. Figures show the mean ±1 SD for each component of the CFF/NFB network for cases exhibiting both wildtype and *ein3;eil1* mutant growth responses (1344 parameters sets). Components include: **(A)** early signaling components (wildtype conditions), **(B)** components downstream of EIN2 (wildtype conditions), and **(C)** negative feedback components affecting growth response (*ein3;eil1* mutant conditions). Black regions in **(B,C)** indicate the mean ±1 standard deviation of growth. Dashed vertical lines indicate time points at which ethylene was introduced (green) and removed (red).

In Figure 5, we plot the time dependence of each network component. This provides insight into how the feedforward and feedback loops shape growth response dynamics. For each component, we plot the mean response of the 1344 parameter sets exhibiting both 2-PGI and MPGR behavior. We also display the standard deviation about the mean (shaded regions) to characterize the heterogeneity of the response. Analogous results for parameter sets exhibiting only 2-PGI or MPGR behavior are provided in Supplemental Figures S3, S4.

The response of the linear portion of the signaling cascade is identical for the wildtype (shown in Figure 5A) and *ein3;eil1* mutant (not shown) topologies upon addition of ethylene. In response to the addition of ethylene at 1 h, the concentration of unbound receptors rapidly declines to levels near zero. This results in a decrease of CTR1 from a relatively high pre-ethylene concentration to a much lower concentration. Following this, EIN2 is no longer inhibited by active CTR1 and rapidly increases in concentration. There is a slight delay in the EIN2 response to ethylene due to the time required for the signal to propagate through the upstream components of the linear signaling cascade. Upon removal of ethylene at 3 h in the wildtype response, components return to their pre-ethylene levels.

The remaining network connections differentiate the wildtype response from the *ein3;eil1* mutant response. Components of the wildtype network are shown in Figure 5B. Increasing EIN2 concentration acts to inhibit both growth and EBF, which is part of the indirect feedforward loop. The direct inhibitory effect of EIN2 on growth is responsible for the first phase of growth inhibition. In response to decreasing EBF concentration, EIN3 concentration increases from an initially low value approximately 30 min after ethylene is introduced. Once EIN3 reaches a sufficiently high concentration, a pronounced second phase of growth inhibition begins. Thus, the coherent feedforward loop leads to the desired 2-PGI.

The effect of the negative feedback loop can be understood by examining the dynamics of growth and GA. For the wildtype topology, GA is inhibited by both EIN3 and growth. A limited increase in GA levels accompanies the first phase of growth inhibition, and is driven by decreasing growth rates. During the second phase of growth inhibition, increasing EIN3 concentration inhibits GA, with EIN3 inhibition outcompeting the effect of decreasing growth rate. This drives GA to a low concentration, minimizing the effect of the negative feedback loop on growth. Thus, in the presence of increased EIN3, the effects of the negative feedback loop are suppressed. In the *ein3;eil1* mutant, however, the inhibitory action of EIN3 on the negative feedback loop is lost. Thus, the negative feedback loop plays a more prominent role since GA is not inhibited by EIN3 (Figure 5C). Additionally, the indirect path of growth inhibition is removed, eliminating the second phase of growth inhibition. Figure 5C shows the response of key network components under these conditions. When increasing EIN2 levels cause a decrease in growth, GA levels increase in response, promoting growth and leading to partial growth recovery. This illustrates the importance of the negative feedback loop for partial growth recovery in the *ein3;eil1* mutant.

After the removal of ethylene at 3 h, it is interesting to note that the average growth rate does not exhibit a large overshoot compared with pre-ethylene levels (Figure 5B). When analyzing individual parameter sets, none of the responses exhibit an overshoot that exceeds pre-ethylene levels by more than 10%, only 5 of 1344 exhibit >5% overshoot, and only 44 of 1344 exhibit >1% overshoot. This is in contrast with experimental results and suggests that modifications of the network or additional components might be needed to adequately capture the overshoot behavior. However, as discussed above, our results show that the core CFF/NFB topology generates key features of the 2-PGI and MPGR responses.

#### 3.1.2. Analysis of parameter sets

**Figure 6:**
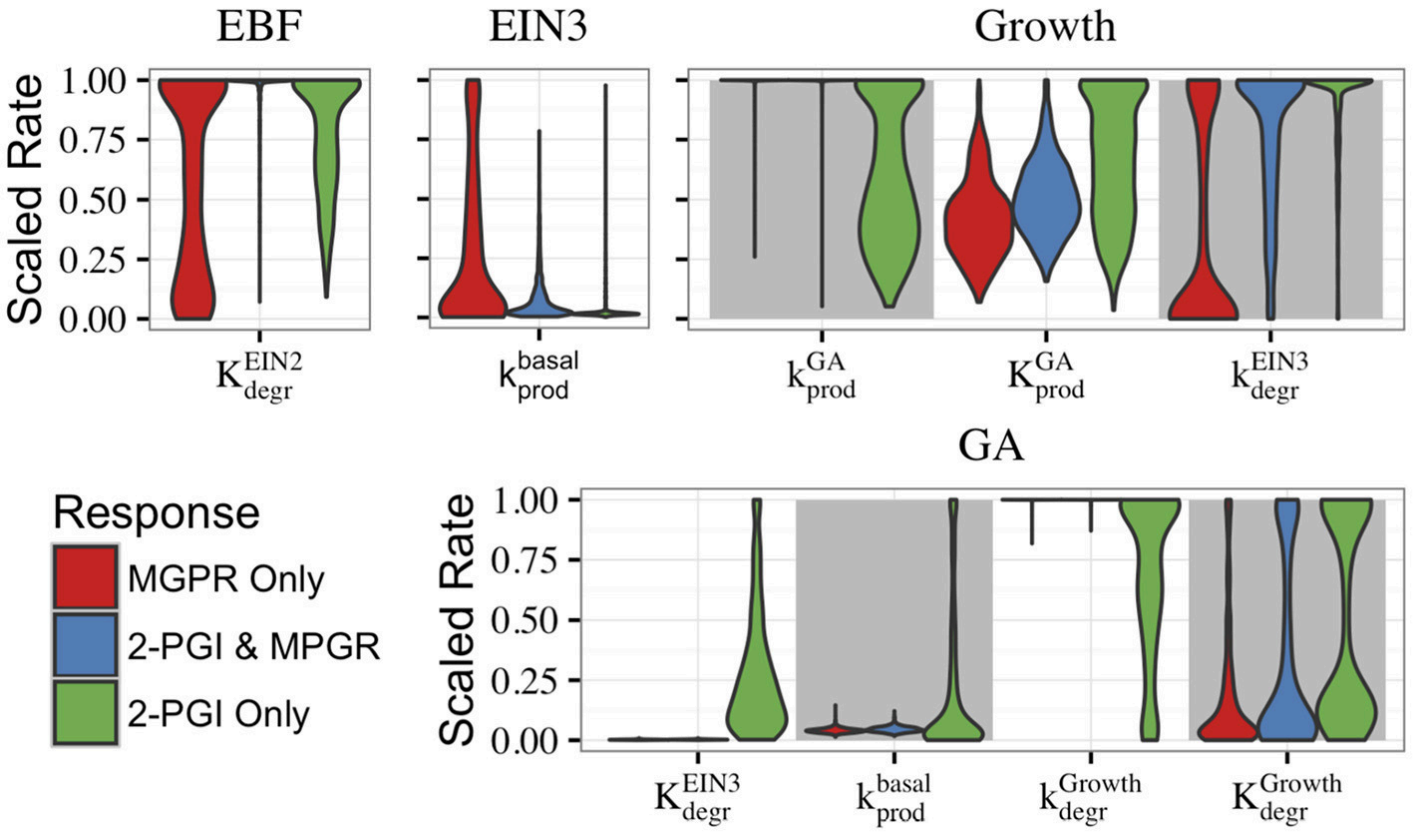
**Distributions of parameters from evolved sets of CFF/NFB network parameters**. Parameter sets exhibiting different combinations of responses are shown. Parameter labels K and k indicate activation coefficients and rate constants, respectively, that are associated with the ODEs governing the species labeled above each figure. Subscripts indicate if the parameter regulates degradation (degr) or production (prod) and superscripts indicate the network component regulating the reaction. Basal rates indicate that the parameter is not regulated by another network component. All parameters were unit normalized using range rescaling.

The roles of the feedforward and feedback loops can be further understood by examining evolved parameters. In particular, it is instructive to characterize the distributions of evolved parameter values for the CFF/NFB network, as certain parameters are constrained to small ranges or excluded from certain parameter regimes. In Figure 6, we compare the distributions of select parameters when the evolved parameter sets are categorized by their behavior (both 2-PGI and MPGR, only 2-PGI, or only MPGR). The distributions of all parameters are shown in Supplemental Figure S5. Parameter values are scaled by normalizing the maximum value to one, and the width of each distribution is scaled such that the maximum width is equal in each distribution. Comparing the distributions for specific parameters highlights key network features leading to each response.

The parameter distributions in Figure 6 provide additional evidence that the feedforward loop plays a key role in generating 2-PGI. The activation coefficient for EIN2-regulated growth degradation of EBF ($K_{\text{degr}}^{\text{EIN2}}$) is excluded from low values in evolved parameter sets exhibiting 2-PGI. As a consequence, EIN2 concentration must reach high levels to significantly inhibit EBF, which contributes to a delay before the second phase of growth inhibition. Interestingly, this parameter is most significantly constrained in evolved parameter sets exhibiting both 2-PGI and MPGR. This is in contrast with the broader distributions seen for the cases exhibiting only one of the targeted responses. Additionally, in parameter sets exhibiting 2-PGI, the rate constant associated with basal production of EIN3 ($k_{\text{prod}}^{\text{basal}}$) occurs at low values. This also contributes to a time delay in the feedforward loop, which is needed for a second phase of growth inhibition.

It is also informative to consider the parameters governing the negative feedback loop (Figure 6). Parameter sets exhibiting MPGR have highly restricted ranges associated with the rate constant for basal production of GA and the parameters for GA inhibition by growth. These restrictions lead to low pre-ethylene levels of GA and a reasonable response of GA as growth declines following ethylene exposure. In cases exhibiting MPGR, the rate constant governing promotion of growth by GA is also restricted to high values and the activation coefficient for the promotion of growth by GA is excluded from the lowest values. Thus, a moderate increase in GA concentration will result in a significant increase in growth. However, excluding the activation coefficient from low values prevents increases from occurring with small changes in GA. Thus, tight regulation of parameters of the negative feedback loop is most readily apparent in parameter sets exhibiting MPGR. This further suggests the importance of the negative feedback loop for MPGR.

#### 3.1.3. Ethylene dose response

**Figure 7:**
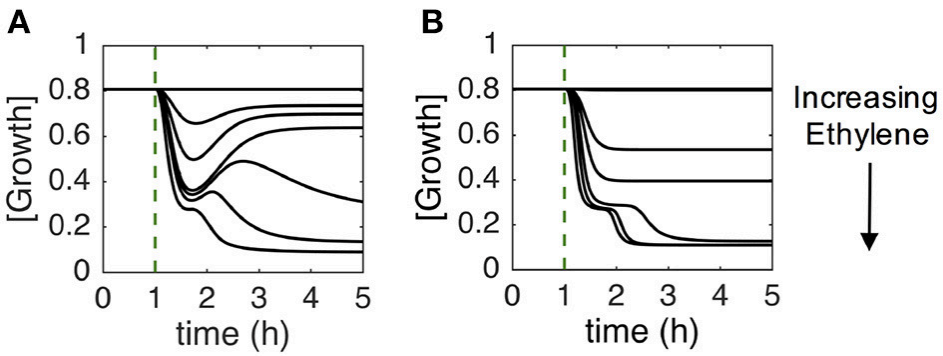
**Characteristic growth responses at sub-saturating ethylene doses**. Parameter sets exhibiting both 2-PGI and MPGR were screened for partial growth recovery to sustained sub-saturating ethylene doses (SSGR). Figures show typical growth responses for parameter sets: **(A)** passing SSGR screening and **(B)** failing SSGR screening.

Given that our network parameters were evolved to target only 2-PGI and MPGR behavior, we were interested in whether other experimentally observed behavior emerged as well. As such, we examined the sub-saturating ethylene dose-response behavior of evolved parameter sets exhibiting both 2-PGI and MPGR. Figure 7A shows an example of sub-saturating ethylene growth recovery (SSGR) that passes our screening criteria. Figure 7B shows an example of a typical growth response failing to exhibit SSGR behavior. Here, there is no growth recovery observed at any ethylene concentration. Of the evolved parameter sets exhibiting both 2-PGI and MPGR, 26% also exhibit SSGR. Partial growth recovery at large sub-saturating ethylene concentrations was observed experimentally (e.g., at 100 ppb in Figure 2C) but was not observed in the CFF/NFB network model. However, the observed adaptive behavior occurring at lower ethylene concentrations is qualitatively consistent with experiments. This emergent property provides additional support for the proposed CFF/NFB network topology.

**Figure 8:**
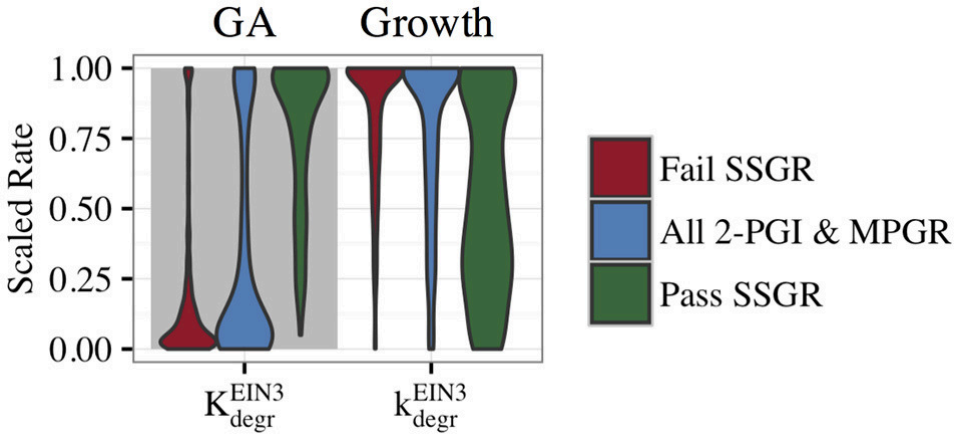
**Distributions of parameters from the CFF/NFB network screened for SSGR behavior**. A comparison of parameter distributions passing and failing SSGR screening (screened parameter sets exhibit both 2-PGI and MPGR).

To gain insight into features that lead to SSGR, we compared the parameter distributions that passed SSGR screening to those that failed SSGR screening. Surprisingly, this revealed nearly identical parameter distributions except for the activation coefficient for GA inhibition by EIN3 and the rate constant associated with inhibition of growth by EIN3 (Figure 8 and Supplemental Figure S6). The activation coefficient regulating GA inhibition by EIN3 occurs at higher values in sets producing SSGR. The rate constant for growth inhibition by EIN3 occurs at lower values more frequently in cases giving SSGR. The distributions of these parameters across all evolved sets exhibiting 2-PGI and/or MPGR are shown in Figure 6. Higher values of the activation coefficient for GA inhibition by EIN3 are found primarily in parameter sets exhibiting 2-PGI, while lower values of the rate constants for inhibition of growth by EIN3 occur primarily in parameter sets exhibiting MPGR. These restrictions apply to the regulation of EIN3 on components of the negative feedback loop. This again suggests that inhibition of the negative feedback loop by the coherent feedforward loop may play a key role in ethylene signaling.

#### 3.1.4. Simplified CFF/NFB networks

**Figure 9:**
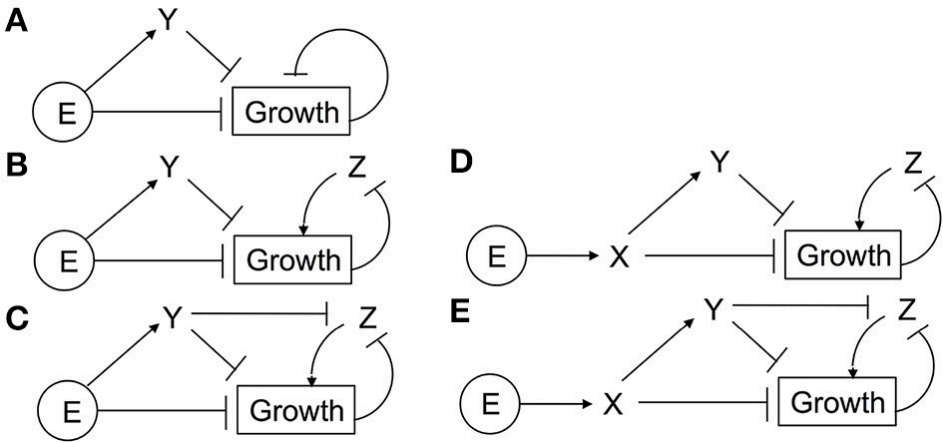
**Simplified CFF/NFB networks**. Simplified networks tested. **(A–E)** Only networks **(D,E)** exhibit both 2-PGI and MPGR.

We have shown that the CFF/NFB network can produce multiple experimentally-observed features of Arabidopsis growth responses to ethylene. Using this network topology as a guide, we probed simplified networks containing coherent feedforward and negative feedback motifs. The networks explored are shown in Figure 9. Ethylene (E) acts as either the first node of the coherent feedforward loop (Figures 9A–C) or as a direct input into the first node of the loop (Figures 9D,E). Additionally, EBF and EIN3 are combined into a single node (Y) which acts to inhibit growth. In the simplest network (Figure 9A), we remove the GA node and allow growth to directly inhibit its own production. For the remaining networks, the role of GA in the negative feedback loop is performed by node Z. To probe the inhibition of the negative feedback loop by the coherent feedforward loop, we tested network topologies with and without the inhibition of Z by Y. Approximately 500 independent optimization runs were performed for each simplified network topology. *Ein3;eil1* mutants were simulated by eliminating node Y. Evolved parameter sets were screened for 2-PGI and MPGR responses and a summary of results are shown in Table 1.

**Table 1:** **Screening results for ethylene growth responses of simplified CFF/NFB networks**.

**Network**	**Total**	**2-PGI & MPGR**	**2-PGI only**	**MPGR only**
A	504	0	0	0
B	500	0	2	0
C	500	0	80	18
D	499	5	1	3
E	500	92	73	37

The simplest network (Figure 9A) failed to produce any parameter sets passing 2-PGI or MPGR screening procedures. Examining the dynamical response of evolved parameter sets revealed two phases of growth inhibition that occurred too early and above the desired growth range. Additionally, no growth recovery was observed upon removal of Y. The addition of node Z to the negative feedback loop (Figure 9B) produced two parameter sets exhibiting 2-PGI but none showing MPGR. When the inhibition of Z by Y is included (Figure 9C), we begin to observe substantial numbers of parameter sets exhibiting either 2-PGI or MPGR. However, no parameter sets simultaneously produced both responses. 2-PGI and MPGR were observed together only when ethylene promoted the first node of the coherent feedforward cascade, which more closely mimics the initial linear signaling cascade. In networks in which Z is not directly inhibited by Y (Figure 9D), 1.0% of parameter sets exhibit both 2-PGI and MPGR responses. When Y regulates Z (Figure 9E), 18.4% of parameter sets exhibit both targeted growth responses. These results suggest the importance of (i) the initial linear cascade in achieving proper timing of growth inhibition and (ii) the inhibition of negative feedback by the coherent feedforward loop in expanding the parameter space in which 2-PGI and MPGR are observed.

### 3.2. EIN2 cleavage with positive feedback

**Figure 10:**
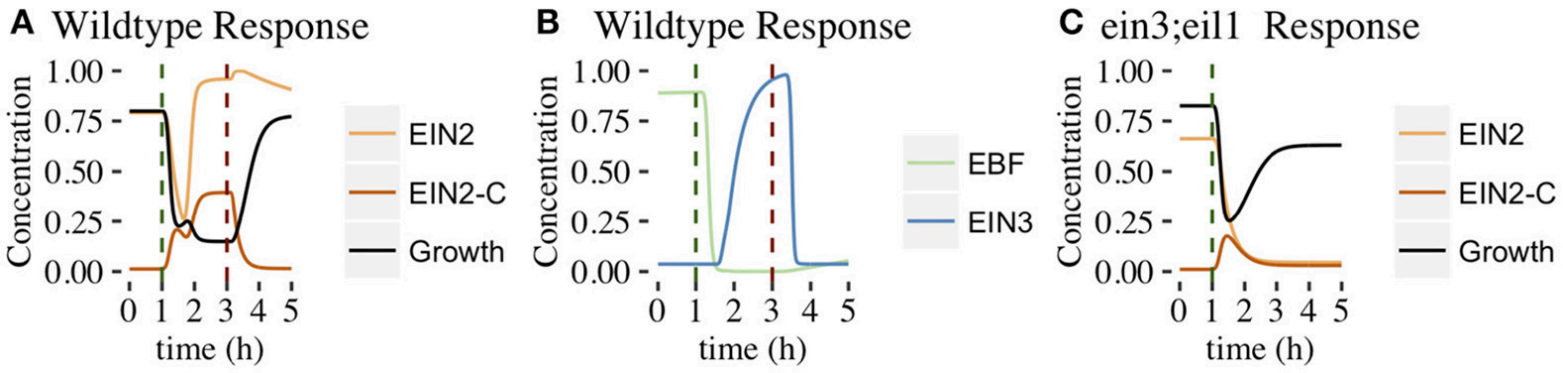
**Time evolution of PFB network components**. Figures show the behavior of the best-performing evolved parameter set that passed 2-PGI, MPGR, and SSGR screening. **(A)** Response of growth, EIN2, and EIN2-C (wildtype conditions). **(B)** Response of components in the positive feedback loop (wildtype conditions). **(C)** Response of growth, EIN2, and EIN2-C (*ein3;eil1* mutant conditions).

In this section, we consider the second proposed network topology with EIN2 cleavage and a positive feedback loop (PFB network, Figure 3B). The network was evolved with the same target responses as before. We obtained evolved parameter sets that produced both 2-PGI and MPGR behavior, but the number was substantially lower than in the CFF/NFB network. Out of 1247 independent runs of the EA, only 5 evolved parameter sets produce both 2-PGI and MPGR. Parameter sets exhibiting only 2-PGI were also uncommon (3 sets). However, a significant proportion of parameter sets exhibited only MPGR (726 sets). Two of the 5 parameter sets exhibiting both 2-PGI and MPGR also display sub-saturating ethylene growth response (SSGR). The limited number of parameter sets exhibiting both targeted responses precludes analysis of parameter distributions. However, studying the dynamic response of the best-performing parameter set exhibiting 2-PGI, MPGR, and SSGR provides valuable insight (Figure 10). Results are representative of the other parameter sets exhibiting 2-PGI and MPGR. Complete results of evolved sets exhibiting 2-PGI, MPGR, and SSGR are presented in the Supplemental Figure S7.

#### 3.2.1. Dynamical response of network components

As in the CFF/NFB network, the introduction of ethylene decreases CTR1 levels (Figure 10A). In the PFB network, the cleavage of EIN2 is no longer inhibited and EIN2-C is produced (Figure 10A). As EIN2-C increases in concentration it inhibits both growth and EBF (Figures 10A,B). EIN2 levels drop during this phase of network response as basal production of EIN2 cannot compensate for the rapid conversion of EIN2 to EIN2-C. EIN2 reaches low concentrations, limiting the resources available for production of EIN2-C. This leads to a transient decline in EIN2-C, which causes the plateau-like region of growth inhibition. Within the feedback loop, lower EBF levels decrease the inhibition of EIN3, which rises and promotes production of EIN2. The rapid rise of EIN2 provides more resources for EIN2-C production. This leads to the second phase of growth inhibition.

Within the *ein3;eil1* mutant, the positive feedback loop is absent. The addition of ethylene leads to EIN2 being converted to EIN2-C, resulting in a decline of EIN2. Without the positive feedback loop, there is no mechanism to further increase EIN2 production and its concentration monotonically decreases. EIN2-C initially increases but then declines as basal degradation eventually dominates the low rates of EIN2-C production associated with low levels of EIN2. As EIN2-C concentration decreases, partial growth recovery is observed (Figure 10C).

#### 3.2.2. Simplified PFB network

**Figure 11:**
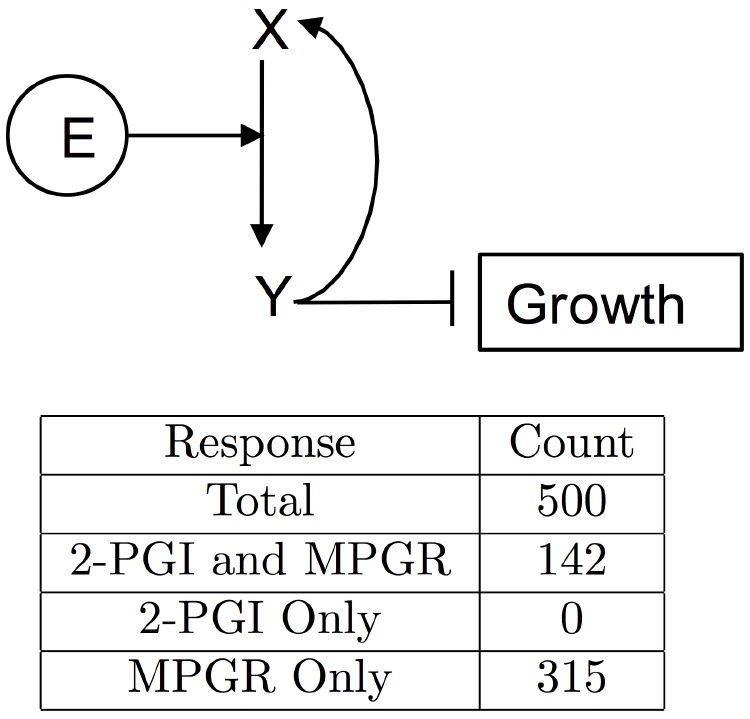
**Simplified PFB network**. Network diagram of the minimal PFB network. The table enumerates results of growth-response screening for evolved parameter sets.

We again explored a simplified network topology that keeps key features of the PFB network. We found that a four component network in which ethylene directly promotes the conversion of X (EIN2) to Y (EIN2-C) can produce both 2-PGI and MPGR (Figure 11). In the network, Y directly inhibits growth and promotes the production of X. We performed 500 optimizations of this network and obtained 142 evolved parameter sets exhibiting both 2-PGI and MPGR. The marked increase in the fraction of parameter sets exhibiting both 2-PGI and MPGR suggests parameter evolution in the full PFB network is hindered by interactions in the positive feedback loop. Three parameters regulate the positive feedback from Y to X in the simplified network, while 9 parameters govern the positive feedback loop in the full network (associated with interactions between EIN2-C, EBF, EIN3, and EIN2). This apparently makes it difficult for our EA to evolve large numbers of parameter sets producing both 2-PGI and MPGR. The large fraction of simplified PFB networks exhibiting both 2-PGI and MPGR again provides support for EIN2 cleavage with positive feedback as a viable network topology.

## 4. Conclusions

We used computational methods to explore hypothesized network topologies underlying ethylene signaling responses in Arabidopsis. We focused on two core networks that are topologically distinct. Using an evolutionary algorithm to explore parameter space, we showed that both network topologies can produce dynamical responses consistent with experimental time-dependent growth data. The core topologies are (i) a coherent feedforward loop that inhibits growth and a negative feedback from growth onto itself (CFF/NFB), and (ii) a network in which ethylene promotes the cleavage of EIN2, with the product of the cleavage inhibiting growth and promoting the production of EIN2 through a positive feedback loop (PFB).

For the CFF/NFB network, high-throughput use of the evolutionary algorithm led to a large number of parameter sets producing responses consistent with experimental growth kinetics under various conditions and genotypes. The results emphasize the importance of various network features for regulating dynamic responses. For example, the two branches of the coherent feedforward loop collectively produce two-phase growth inhibition (2-PGI), and the negative feedback loop is critical for mutant partial growth recovery (MPGR). Our study additionally suggests that 2-PGI and MPGR coexist in a broader parameter regime when the negative feedback loop is suppressed by an intermediate component of the coherent feedforward cascade. The large number of parameter sets producing 2-PGI and MPGR behavior provide insight into important regimes of parameter space. Additionally, a large fraction of these parameter sets also exhibit sub-saturating ethylene growth response (SSGR), even though this was not a targeted response by the evolutionary algorithm. Taken together, these results provide support for the CFF/NFB network as a viable network topology underlying ethylene signaling.

For the PFB network, the evolutionary algorithm led to far fewer parameter sets producing both 2-PGI and MPGR behavior, yet the dynamics of their responses provided insight into the mechanisms underlying the network topology. A key feature of the network is that EIN2 is converted to EIN2-C and its transient depletion upon the addition of ethylene is responsible for the plateau phase of growth inhibition. Two of the evolved parameter sets also exhibited SSGR, indicating that this emergent behavior is also possible in the PFB network. Although we generated far more parameter sets producing 2-PGI and MPGR for the CFF/NFB network, this does not necessarily imply that it is biologically more likely. For example, the region of parameter space for the PFB network that gives the desired behavior may be smaller or more difficult to identify with our EA, but this does not exclude the PFB network as biologically feasible.

It is interesting to note that different plant species have qualitatively different ethylene response kinetics (Kim et al., 2012). For example, some plant species (millet) have only a transient first phase response and some (rice) have only a prolonged second phase response. The paper by Kim et al. first proposed the CFF/NFB network studied here. For millet, Kim et al. proposed that the circuit controlled by EIN3/EIL1 was missing to give the transient response; for rice, it was proposed that the rapid, EIN3/EIL1-independent output of EIN2 is missing. The first case was analyzed in this paper when we analyzed the MPGR response. An interesting feature of the CFF/NFB model is that there is a simple conceptual way to modify the network to generate responses consistent with other species. It is less clear how the PFB network could be modified in an analogous manner to generate growth response kinetics consistent with the rice and millet studies. Further exploration of the network topology across species is an interesting area for future exploration.

Even though both models exhibited SSGR behavior that was similar to what has been observed experimentally, the kinetics of the computational responses are subtly different from experimental observations. In particular, there was no long-time recovery at high sub-saturating ethylene concentrations and incomplete recovery at low concentrations. It has been suggested that responses to low levels of ethylene are in large part a result of receptor clustering, where ligand occupancy of one receptor affects the signaling state of surrounding receptors through direct interactions and results in signal amplification at low ethylene levels (Gamble et al., 2002; Binder and Bleecker, 2003; Binder et al., 2004b). Computational models invoking receptor clustering indicate this element can affect both sensitivity and adaptation (Bray et al., 1998). Our models did not incorporate this feature, which would likely affect features of the SSGR. Additionally, our models do not incorporate spatial information. For instance, it is now known that EIN2-C translocates to the nucleus to affect ethylene signaling (Ju et al., 2012; Qiao et al., 2012; Wen et al., 2012). Cleavage of EIN2 was not incorporated into the CFF/NFB network and translocation of EIN2-C was not explicitly incorporated into either model. This translocation also may have diverse functions since it has recently been found that EIN2-C in the cytosol also has a role in ethylene signaling (Li et al., 2015; Merchante et al., 2015). It is likely that spatial changes in important components such as EIN2-C have a role in adaptation.

Despite these differences, our calculations show that several simple networks can recapitulate the ethylene growth responses observed experimentally. The dynamic responses observed provide opportunities for experimental exploration. A comparison of the dynamical response of individual components for each network is shown in Supplemental Figure S7. For example, the PFB network shows that when ethylene is added there is a transient decrease in EIN2 levels followed by accumulation of EIN2. By contrast, the CFF/NFB model predicts qualitatively different accumulation kinetics for EIN2, with no transient decrease. Thus, one avenue of experimentation can be to obtain more detailed spatio-temporal information about the accumulation of EIN2 (and EIN2-C) to determine if the details predicted by the calculations in either model occur when saturating levels of ethylene are added. For example, a detailed time-course of EIN2-C accumulation or EIN2 full-length protein is lacking. Such information would help determine which, if either, model correctly predicts the accumulation pattern for EIN2. Additionally, removing EIN3 from the CFF/NFB model has minimal effect on the time-course of EIN2 accumulation when ethylene is added, but has a profound effect on both EIN2 and EIN2-C levels in the PFB model. Thus, experiments examining EIN2 and EIN2-C levels in *ein3;eil1* double mutants would also be informative. Another example is the involvement of GA in the CFF/NFB network where it plays a larger role in the growth kinetics observed in the *ein3;eil1* mutants. Detailed information about changes in GA levels would provide a test of this model and whether the negative feedback loop needs to be incorporated into the PFB network. Such experimental details will help determine which network topology, if either, could serve as the ethylene signaling transduction network of Arabidopsis. It is also possible that a combination of the two models or different network topologies will yield emergent properties that are closer to experimental observations. Additional experimental details about the spatio-temporal changes that occur in each component of the pathway will allow us to refine the above models or develop additional network topologies.

In summary, these calculations show that a basic mechanistic understanding of ethylene growth response and recovery kinetics is possible without detailed knowledge of the molecular mechanisms or enzymatic kinetic parameters. Given that ethylene signal transduction has been highly studied for several decades, we anticipate that major advances in our understanding about this pathway will be to provide details about network interactions, reaction kinetics, and changes in the spatial distribution of proteins in the pathway. Our hope is that with more refined experimental input, we can refine the network models to provide insights into how plants respond to ethylene.

## Author contributions

AP, BB, and SA designed research. AP, FM, and BE performed research. AP, BB, and SA analyzed data and wrote the paper.

## Funding

This work was funded by NSF Grants (IOS-1254423, MCB-1517032) to BB.

### Conflict of interest statement

The authors declare that the research was conducted in the absence of any commercial or financial relationships that could be construed as a potential conflict of interest.
