# Supplementary material for: Analysis of Network Topologies Underlying Ethylene Growth Response Kinetics
Source: Front Plant Sci. 2016 Aug 30;7:1308. doi: 10.3389/fpls.2016.01308 (PMC5003821; doi:10.3389/fpls.2016.01308)
Supplement: Supplementary file 1 [file Presentation1.pdf]

Supplementary Material: Analysis of Network Topologies Underlying Ethylene Growth Response Kinetics

Aaron M. Prescott, Forest W. McCullough, Bryan L. Eldreth, Brad M. Binder* and Steven M. Abel*

*Correspondence:
Email:
bbinder@utk.edu, abel@utk.edu

1 SIGNALING NETWORKS: ORDINARY DIFFERENTIAL EQUATIONS

1.1 Description of notation

Shown below are the ordinary differential equations describing the time evolution of each network considered in the paper. Square brackets denote the concentration of a network component. We use the following notation for network parameters: k indicates a reaction rate, K indicates an activation coefficient appearing in a Hill equation, and N indicates a Hill coefficient. The subscripts "prod' and "degr" indicate whether a rate constant is associated with production or degradation; an associated superscript refers to the relevant network component. Remaining terms appear within large parentheses like the following:

$$-\left(k_{\text{degr}}\frac{[\text{CTR1}]^N}{K_{\text{degr}}^N+[\text{CTR1}]^N}\right)_{\text{CTR1}}[\text{EIN2}] \tag{S1}$$

This term is associated with the degradation of EIN2 in the CFF/NFB network. Each of the three parameters within the parentheses has two implied associations: Each represents the effect of CTR1 (subscript of parentheses) on the degradation of EIN2 (term being modified by the factor in the parentheses). In other words, the subscript outside of the parentheses indicates the network component that regulates the interaction.

1.2 Complete CFF/NFB Network

$$\frac{d[\mathrm{R}]}{dt} = k^{\mathrm{R}}_{\mathrm{prod}}(1 - [\mathrm{R}]) - k^{\mathrm{E}}_{\mathrm{degr}}[\mathrm{E}][\mathrm{R}] \tag{S2}$$

$$\frac{d[\mathrm{CTR1}]}{dt} = \left(k_{\mathrm{prod}}\frac{[\mathrm{R}]^N}{K^N_{\mathrm{prod}} + [\mathrm{R}]^N}\right)_{\mathrm{R}}(1 - [\mathrm{CTR1}]) - k^{\mathrm{CTR1}}_{\mathrm{degr}}[\mathrm{CTR1}] \tag{S3}$$

$$\frac{d[\mathrm{EIN2}]}{dt} = k^{\mathrm{EIN2}}_{\mathrm{prod}}(1 - [\mathrm{EIN2}]) - \left(k_{\mathrm{degr}}\frac{[\mathrm{CTR1}]^N}{K^N_{\mathrm{degr}} + [\mathrm{CTR1}]^N}\right)_{\mathrm{CTR1}}[\mathrm{EIN2}] \tag{S4}$$

$$\frac{d[\mathrm{EBF}]}{dt} = k^{\mathrm{EBF}}_{\mathrm{prod}}(1 - [\mathrm{EBF}]) - \left(k_{\mathrm{degr}}\frac{[\mathrm{EIN2}]^N}{K^N_{\mathrm{degr}} + [\mathrm{EIN2}]^N}\right)_{\mathrm{EIN2}}[\mathrm{EBF}] \tag{S5}$$

$$\frac{d[\mathrm{EIN3}]}{dt} = k^{\mathrm{EIN3}}_{\mathrm{prod}}(1 - [\mathrm{EIN3}]) - \left(k_{\mathrm{degr}}\frac{[\mathrm{EBF}]^N}{K^N_{\mathrm{degr}} + [\mathrm{EBF}]^N}\right)_{\mathrm{EBF}}[\mathrm{EIN3}] \tag{S6}$$

$$\frac{d[\mathrm{GA}]}{dt} = k^{\mathrm{GA}}_{\mathrm{prod}}(1 - [\mathrm{GA}]) - \left(k_{\mathrm{degr}}\frac{[\mathrm{EIN3}]^N}{K^N_{\mathrm{degr}} + [\mathrm{EIN3}]^N}\right)_{\mathrm{EIN3}}[\mathrm{GA}] \tag{S7}$$

$$- \left(k_{\mathrm{degr}}\frac{[\mathrm{Growth}]^N}{K^N_{\mathrm{degr}} + [\mathrm{Growth}]^N}\right)_{\mathrm{Growth}}[\mathrm{GA}] \tag{S8}$$

$$\frac{d[\mathrm{Growth}]}{dt} = k^{\mathrm{Growth}}_{\mathrm{prod}}(1 - [\mathrm{Growth}]) + \left(k_{\mathrm{prod}}\frac{[\mathrm{GA}]^N}{K^N_{\mathrm{prod}} + [\mathrm{GA}]^N}\right)_{\mathrm{GA}}(1 - [\mathrm{Growth}]) \tag{S9}$$

$$- \left(k_{\mathrm{degr}}\frac{[\mathrm{EIN2}]^N}{K^N_{\mathrm{degr}} + [\mathrm{EIN2}]^N}\right)_{\mathrm{EIN2}}[\mathrm{Growth}] - \left(k_{\mathrm{degr}}\frac{[\mathrm{EIN3}]^N}{K^N_{\mathrm{degr}} + [\mathrm{EIN3}]^N}\right)_{\mathrm{EIN3}}[\mathrm{Growth}] \tag{S10}$$

1.3 Simplified CFF/NFB Network (A)

$$\frac{d[\mathrm{Y}]}{dt} = \left(k_{\mathrm{prod}}\frac{[\mathrm{E}]^N}{K^N_{\mathrm{prod}} + [\mathrm{E}]^N}\right)_{\mathrm{E}}(1 - [\mathrm{Y}]) - k^{\mathrm{Y}}_{\mathrm{degr}}[\mathrm{Y}] \tag{S11}$$

$$\frac{d[\mathrm{Growth}]}{dt} = k^{\mathrm{Growth}}_{\mathrm{prod}}(1 - [\mathrm{Growth}]) - \left(k_{\mathrm{degr}}\frac{[\mathrm{E}]^N}{K^N_{\mathrm{degr}} + [\mathrm{E}]^N}\right)_{\mathrm{E}}[\mathrm{Growth}] \tag{S12}$$

$$- \left(k_{\mathrm{degr}}\frac{[\mathrm{Y}]^N}{K^N_{\mathrm{degr}} + [\mathrm{Y}]^n}\right)_{\mathrm{Y}}[\mathrm{Growth}] - \left(k_{\mathrm{degr}}\frac{[\mathrm{Growth}]^N}{K^N_{\mathrm{degr}} + [\mathrm{Growth}]^N}\right)_{\mathrm{Growth}}[\mathrm{Growth}] \tag{S13}$$

1.4 Simplified CFF/NFB Network (B)

$$\frac{d[\mathrm{Y}]}{dt} = \left(k_{\mathrm{prod}}\frac{[\mathrm{E}]^N}{K_{\mathrm{prod}}^N + [\mathrm{E}]^N}\right)_{\mathrm{E}}(1-[\mathrm{Y}]) - k_{\mathrm{degr}}^{\mathrm{Y}}[\mathrm{Y}] \tag{S14}$$

$$\frac{d[\mathrm{Z}]}{dt} = k_{\mathrm{prod}}^{\mathrm{Z}}(1-[\mathrm{Z}]) - \left(k_{\mathrm{degr}}\frac{[\mathrm{Growth}]^N}{K_{\mathrm{degr}}^N + [\mathrm{Growth}]^N}\right)_{\mathrm{Growth}}[\mathrm{Z}] \tag{S15}$$

$$\frac{d[\mathrm{Growth}]}{dt} = k_{\mathrm{prod}}^{\mathrm{Growth}}(1-[\mathrm{Growth}]) + \left(k_{\mathrm{prod}}\frac{[\mathrm{Z}]^N}{K_{\mathrm{prod}}^N + [\mathrm{Z}]^N}\right)_{\mathrm{Z}}(1-[\mathrm{Growth}]) \tag{S16}$$

$$- \left(k_{\mathrm{degr}}\frac{[\mathrm{E}]^N}{K_{\mathrm{degr}}^N + [\mathrm{E}]^N}\right)_{\mathrm{E}}[\mathrm{Growth}] - \left(k_{\mathrm{degr}}\frac{[\mathrm{Y}]^N}{K_{\mathrm{degr}}^N + [\mathrm{Y}]^n}\right)_{\mathrm{Y}}[\mathrm{Growth}] \tag{S17}$$

1.5 Simplified CFF/NFB Network (C)

$$\frac{d[\mathrm{Y}]}{dt} = \left(k_{\mathrm{prod}}\frac{[\mathrm{E}]^N}{K_{\mathrm{prod}}^N + [\mathrm{E}]^N}\right)_{\mathrm{E}}(1-[\mathrm{Y}]) - k_{\mathrm{degr}}^{\mathrm{Y}}[\mathrm{Y}] \tag{S18}$$

$$\frac{d[\mathrm{Z}]}{dt} = k_{\mathrm{prod}}^{\mathrm{Z}}(1-[\mathrm{Z}]) - \left(k_{\mathrm{degr}}\frac{[\mathrm{Y}]^N}{K_{\mathrm{degr}}^N + [\mathrm{Y}]^N}\right)_{\mathrm{Y}}[\mathrm{Z}] - \left(k_{\mathrm{degr}}\frac{[\mathrm{Growth}]^N}{K_{\mathrm{degr}}^N + [\mathrm{Growth}]^N}\right)_{\mathrm{Growth}}[\mathrm{Z}] \tag{S19}$$

$$\frac{d[\mathrm{Growth}]}{dt} = k_{\mathrm{prod}}^{\mathrm{Growth}}(1-[\mathrm{Growth}]) + \left(k_{\mathrm{prod}}\frac{[\mathrm{Z}]^N}{K_{\mathrm{prod}}^N + [\mathrm{Z}]^N}\right)_{\mathrm{Z}}(1-[\mathrm{Growth}]) \tag{S20}$$

$$- \left(k_{\mathrm{degr}}\frac{[\mathrm{E}]^N}{K_{\mathrm{degr}}^N + [\mathrm{E}]^N}\right)_{\mathrm{E}}[\mathrm{Growth}] - \left(k_{\mathrm{degr}}\frac{[\mathrm{Y}]^N}{K_{\mathrm{degr}}^N + [\mathrm{Y}]^N}\right)_{\mathrm{Y}}[\mathrm{Growth}] \tag{S21}$$

1.6 Simplified CFF/NFB Network (D)

$$\frac{d[\mathrm{X}]}{dt} = \left(k_{\mathrm{prod}}\frac{[\mathrm{E}]^N}{K_{\mathrm{prod}}^N + [\mathrm{E}]^N}\right)_{\mathrm{E}}(1-[\mathrm{X}]) - k_{\mathrm{degr}}^{\mathrm{X}}[\mathrm{X}] \tag{S22}$$

$$\frac{d[\mathrm{Y}]}{dt} = \left(k_{\mathrm{prod}}\frac{[\mathrm{X}]^N}{K_{\mathrm{prod}}^N + [\mathrm{X}]^N}\right)_{\mathrm{X}}(1-[\mathrm{Y}]) - k_{\mathrm{degr}}^{\mathrm{Y}}[\mathrm{Y}] \tag{S23}$$

$$\frac{d[\mathrm{Z}]}{dt} = k_{\mathrm{prod}}^{\mathrm{Z}}(1-[\mathrm{Z}]) - \left(k_{\mathrm{degr}}\frac{[\mathrm{Growth}]^N}{K_{\mathrm{degr}}^N + [\mathrm{Growth}]^N}\right)_{\mathrm{Growth}}[\mathrm{Z}] \tag{S24}$$

$$\frac{d[\mathrm{Growth}]}{dt} = k_{\mathrm{prod}}^{\mathrm{Growth}}(1-[\mathrm{Growth}]) + \left(k_{\mathrm{prod}}\frac{[\mathrm{Z}]^N}{K_{\mathrm{prod}}^N + [\mathrm{Z}]^N}\right)_{\mathrm{Z}}(1-[\mathrm{Growth}]) \tag{S25}$$

$$- \left(k_{\mathrm{degr}}\frac{[\mathrm{X}]^N}{K_{\mathrm{degr}}^N + [\mathrm{X}]^N}\right)_{\mathrm{X}}[\mathrm{Growth}] - \left(k_{\mathrm{degr}}\frac{[\mathrm{Y}]^N}{K_{\mathrm{degr}}^N + [\mathrm{Y}]^N}\right)_{\mathrm{Y}}[\mathrm{Growth}] \tag{S26}$$

1.7 Simplified CFF/NFB Network (E)

$$\frac{d[\mathrm{X}]}{dt} = \left(k_{\mathrm{prod}}\frac{[\mathrm{E}]^N}{K_{\mathrm{prod}}^N + [\mathrm{E}]^N}\right)_{\mathrm{E}}(1-[\mathrm{X}]) - k_{\mathrm{degr}}^{\mathrm{X}}[\mathrm{X}] \tag{S27}$$

$$\frac{d[\mathrm{Y}]}{dt} = \left(k_{\mathrm{prod}}\frac{[\mathrm{X}]^N}{K_{\mathrm{prod}}^N + [\mathrm{X}]^N}\right)_{\mathrm{X}}(1-[\mathrm{Y}]) - k_{\mathrm{degr}}^{\mathrm{Y}}[\mathrm{Y}] \tag{S28}$$

$$\frac{d[\mathrm{Z}]}{dt} = k_{\mathrm{prod}}^{\mathrm{Z}}(1-[\mathrm{Z}]) - \left(k_{\mathrm{degr}}\frac{[\mathrm{Y}]^N}{K_{\mathrm{degr}}^N + [\mathrm{Y}]^N}\right)_{\mathrm{Y}}[\mathrm{Z}] - \left(k_{\mathrm{degr}}\frac{[\mathrm{Growth}]^N}{K_{\mathrm{degr}}^N + [\mathrm{Growth}]^N}\right)_{\mathrm{Growth}}[\mathrm{Z}] \tag{S29}$$

$$\frac{d[\mathrm{Growth}]}{dt} = k_{\mathrm{prod}}^{\mathrm{Growth}}(1-[\mathrm{Growth}]) + \left(k_{\mathrm{prod}}\frac{[\mathrm{Z}]^N}{K_{\mathrm{prod}}^N + [\mathrm{Z}]^N}\right)_{\mathrm{Z}}(1-[\mathrm{Growth}]) \tag{S30}$$

$$- \left(k_{\mathrm{degr}}\frac{[\mathrm{X}]^N}{K_{\mathrm{degr}}^N + [\mathrm{X}]^N}\right)_{\mathrm{X}}[\mathrm{Growth}] - \left(k_{\mathrm{degr}}\frac{[\mathrm{Y}]^N}{K_{\mathrm{degr}}^N + [\mathrm{Y}]^N}\right)_{\mathrm{Y}}[\mathrm{Growth}] \tag{S31}$$

1.8 Complete PFB Network

The parameters describing the CTR1-regulated conversion of EIN2 to EIN2-C appear as part of a production term in the EIN2 ODE and as part of a degradation term in EIN2-C. To demarcate these parameters from the remaining parameters that are classified strictly as production or degradation, these

parameters been given the subscript "cat."

$$\frac{d[\mathrm{R}]}{dt} = k_{\mathrm{prod}}^{\mathrm{R}}(1-[\mathrm{R}]) - k_{\mathrm{degr}}^{\mathrm{E}}[\mathrm{E}][\mathrm{R}] \tag{S32}$$

$$\frac{d[\mathrm{CTR1}]}{dt} = \left(k_{\mathrm{prod}}\frac{[\mathrm{R}]^N}{K_{\mathrm{prod}}^N+[\mathrm{R}]^N}\right)_{\mathrm{R}}(1-[\mathrm{CTR1}]) - k_{\mathrm{degr}}^{\mathrm{CTR1}}[\mathrm{CTR1}] \tag{S33}$$

$$\frac{d[\mathrm{EIN2}]}{dt} = k_{\mathrm{prod}}^{\mathrm{EIN2}}(1-[\mathrm{EIN2}]) + \left(k_{\mathrm{prod}}\frac{[\mathrm{EIN3}]^N}{K_{\mathrm{prod}}^N+[\mathrm{EIN3}]^N}\right)_{\mathrm{EIN3}}(1-[\mathrm{EIN2}]) \tag{S34}$$

$$-\left(k_{\mathrm{cat}}\frac{K_{\mathrm{cat}}^N}{K_{\mathrm{cat}}^N+[\mathrm{CTR1}]^N}\right)_{\mathrm{CTR1}}[\mathrm{EIN2}](1-[\mathrm{EIN2\text{-}C}]) \tag{S35}$$

$$\frac{d[\mathrm{EIN2\text{-}C}]}{dt} = \left(k_{\mathrm{cat}}\frac{K_{\mathrm{cat}}^N}{K_{\mathrm{cat}}^N+[\mathrm{CTR1}]^N}\right)_{\mathrm{CTR1}}[\mathrm{EIN2}](1-[\mathrm{EIN2\text{-}C}]) - k_{\mathrm{degr}}^{\mathrm{EIN2\text{-}C}}[\mathrm{EIN2\text{-}C}] \tag{S36}$$

$$\frac{d[\mathrm{EBF}]}{dt} = k_{\mathrm{prod}}^{\mathrm{EBF}}(1-[\mathrm{EBF}]) - \left(k_{\mathrm{degr}}\frac{[\mathrm{EIN2\text{-}C}]^N}{K_{\mathrm{degr}}^N+[\mathrm{EIN2\text{-}C}]^N}\right)_{\mathrm{EIN2\text{-}C}}[\mathrm{EBF}] \tag{S37}$$

$$\frac{d[\mathrm{EIN3}]}{dt} = k_{\mathrm{prod}}^{\mathrm{EIN3}}(1-[\mathrm{EIN3}]) - \left(k_{\mathrm{degr}}\frac{[\mathrm{EBF}]^N}{K_{\mathrm{degr}}^N+[\mathrm{EBF}]^N}\right)_{\mathrm{EBF}}[\mathrm{EIN3}] \tag{S38}$$

$$\frac{d[\mathrm{Growth}]}{dt} = k_{\mathrm{prod}}^{\mathrm{Growth}}(1-[\mathrm{Growth}]) - \left(k_{\mathrm{degr}}\frac{[\mathrm{EIN2\text{-}C}]^N}{K_{\mathrm{degr}}^N+[\mathrm{EIN2\text{-}C}]^N}\right)_{\mathrm{EIN2C}}(1-[\mathrm{Growth}]) \tag{S39}$$

$$\tag{S40}$$

1.9 Simplified PFB Network

$$\frac{d[\mathrm{X}]}{dt} = k_{\mathrm{prod}}^{\mathrm{X}}(1-[\mathrm{X}]) + \left(k_{\mathrm{prod}}\frac{[\mathrm{Y}]^N}{K_{\mathrm{prod}}^N+[\mathrm{Y}]^N}\right)_{\mathrm{Y}}(1-[\mathrm{X}]) - \left(k_{\mathrm{cat}}\frac{[\mathrm{E}]^N}{K_{\mathrm{cat}}^N+[\mathrm{E}]^N}\right)_{\mathrm{E}}[\mathrm{X}](1-[\mathrm{Y}]) \tag{S41}$$

$$\frac{d[\mathrm{Y}]}{dt} = \left(k_{\mathrm{cat}}\frac{[\mathrm{E}]^N}{K_{\mathrm{cat}}^N+[\mathrm{E}]^N}\right)_{\mathrm{E}}[\mathrm{X}](1-[\mathrm{Y}]) - k_{\mathrm{degr}}^{\mathrm{Y}}[\mathrm{Y}] \tag{S42}$$

$$\frac{d[\mathrm{Growth}]}{dt} = k_{\mathrm{prod}}^{\mathrm{Growth}}(1-[\mathrm{Growth}]) - \left(k_{\mathrm{degr}}\frac{[\mathrm{Y}]^N}{K_{\mathrm{degr}}^N+[\mathrm{Y}]^N}\right)_{\mathrm{Y}}[\mathrm{Growth}] \tag{S43}$$

$$\tag{S44}$$

2 PARAMETERS AND SCREENING PROCEDURES

2.1 Parameters used

2.1.1 Fitness function: Target growth values and scaling factors (wildtype conditions)

Elapsed Time (h)	Targ. Growth Rate	α_i
0.42	0.750	3
0.75	0.750	3
1.00	0.750	3
1.17	0.611	2
1.25	0.441	2
1.33	0.339	2
1.42	0.295	2
1.58	0.278	5
1.83	0.268	5
2.25	0.119	5
2.58	0.095	7
3.17	0.119	5
3.50	0.220	2
3.75	0.509	2
4.00	0.754	2
4.17	0.877	2
4.5	0.971	2

2.1.2 Fitness function: Target growth values and scaling factors (*ein3;eil1* mutant conditions)

Elapsed Time (h)	Exp. Growth Rate	β_i
0.17	0.800	2
0.58	0.800	2
1.00	0.800	2
1.33	0.387	1
1.67	0.327	1
1.83	0.309	1
2.00	0.311	1
2.17	0.331	1
2.42	0.363	1
2.67	0.449	1
2.92	0.622	1
3.33	0.692	1
4.08	0.692	2
4.50	0.700	2

2.1.3 Parameter ranges

Parameter	Min	Max
$k_{\text{prod}}, k_{\text{degr}}, k_{\text{cat}}$ (h^{-1})	0	100 #
$K_{\text{prod}}, K_{\text{degr}}, K_{\text{cat}}$	0	1
N	1 or 2 *	6

* The lower limit of 2 was used for the CFF/NFB network; the lower limit of 1 was used for the PFB network.

# This range was used for all k-parameters except for k_{degr} associated with EIN2-regulated EBF degradation in the PFB network. For this one case, 719 of the evolved sets used the full range. We then restricted the remaining 528 cases to sample from the range of 50-100 h^{-1}.

2.2 Logic diagrams used for screening

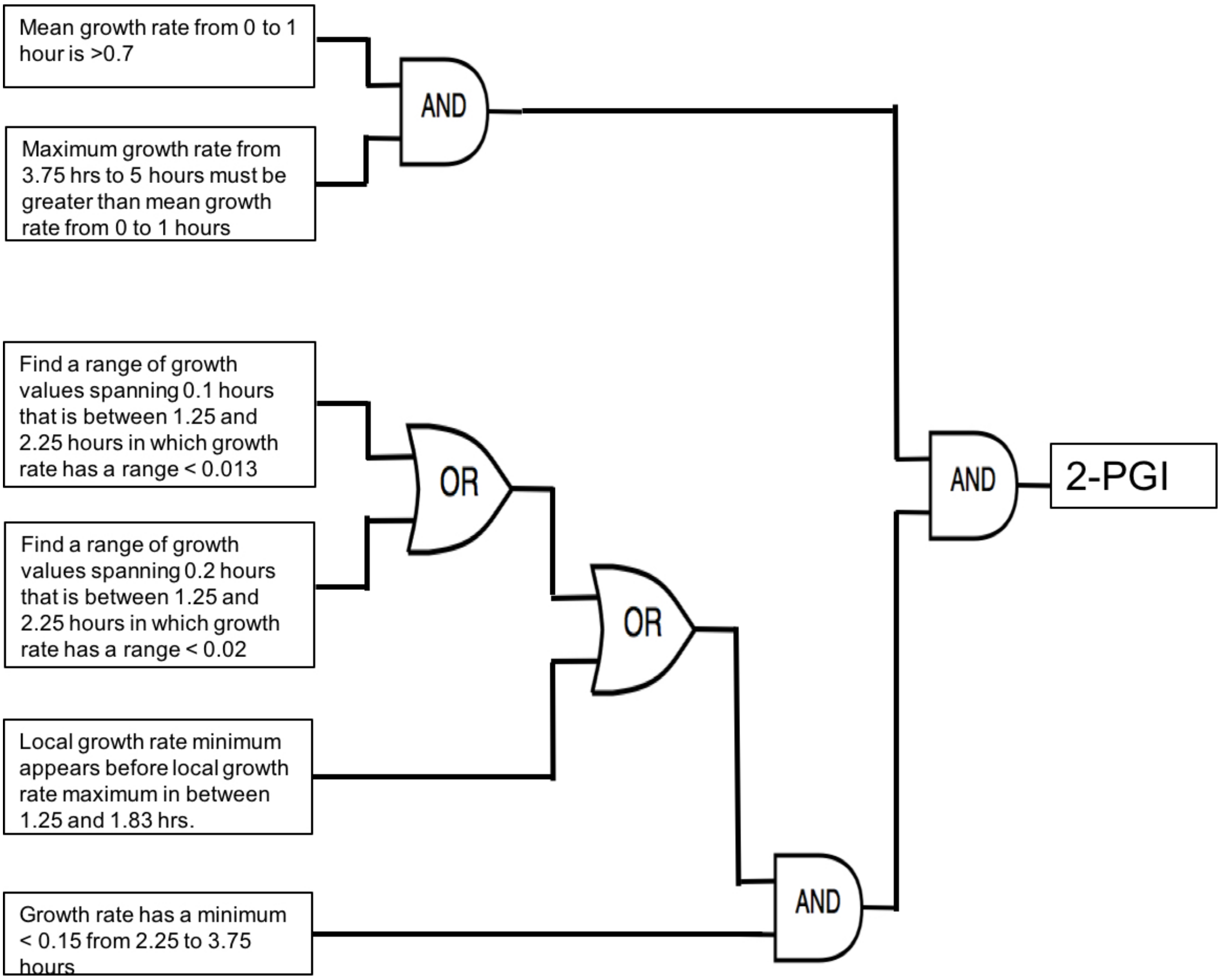

Figure S1. Logic diagram for 2-PGI screening. Growth responses under wildtype saturating ethylene conditions are screened using the outlined logic diagram. Growth responses reaching the final **2-PGI** terminal pass the screening procedure.

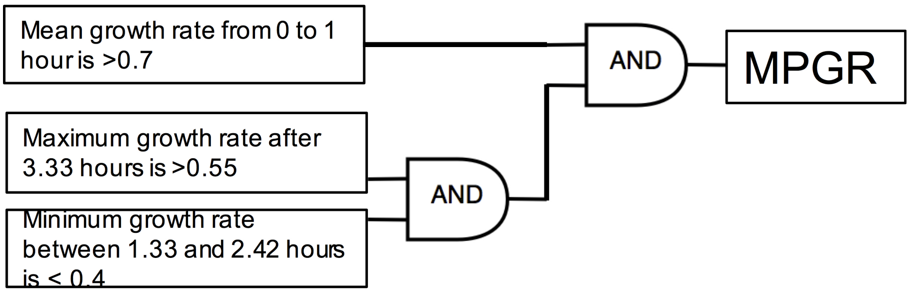

Figure S2. Logic diagram for MPGR screening. Growth responses under *ein3;eil1* mutant saturating ethylene conditions are screened using the outlined logic diagram. Growth responses reaching the final **MPGR** terminal pass the screening procedure.

3 DYNAMICAL RESPONSES AND PARAMETER DISTRIBUTIONS

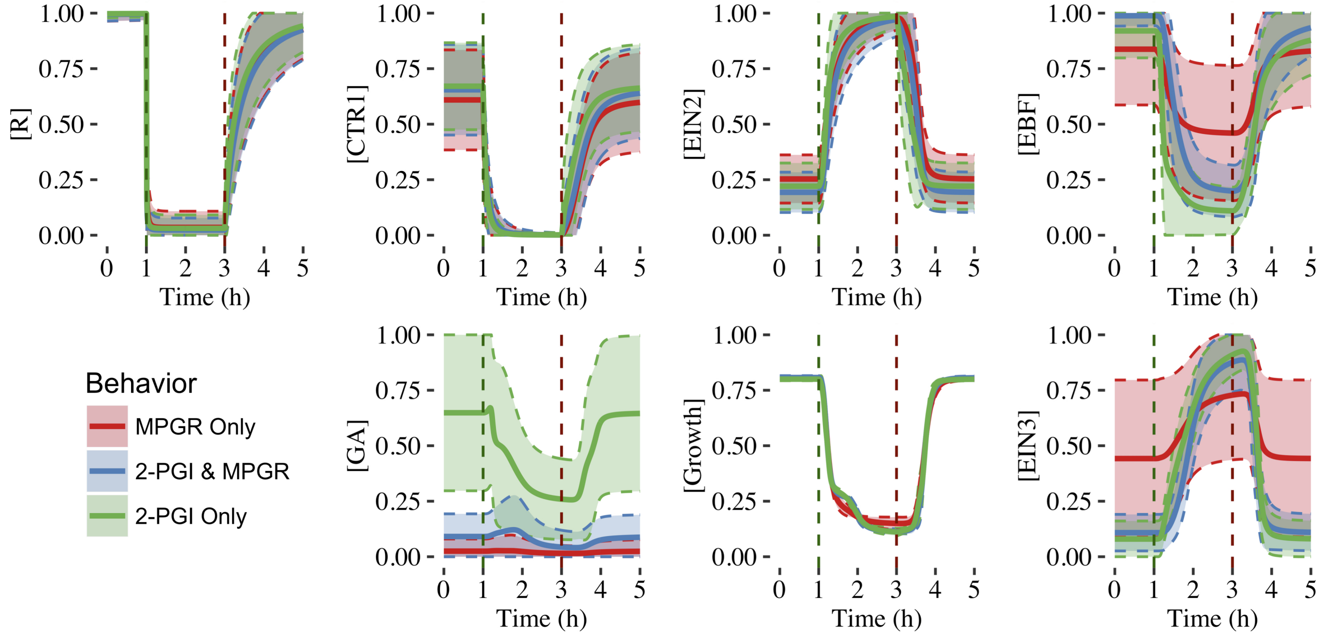

Figure S3. Time evolution of CFF/NFB network components (wildtype conditions). The mean response ± 1 standard deviation for all components from evolved CFF/NFB parameter sets passing 2-PGI and/or MPGR screening under wildtype saturating ethylene conditions.

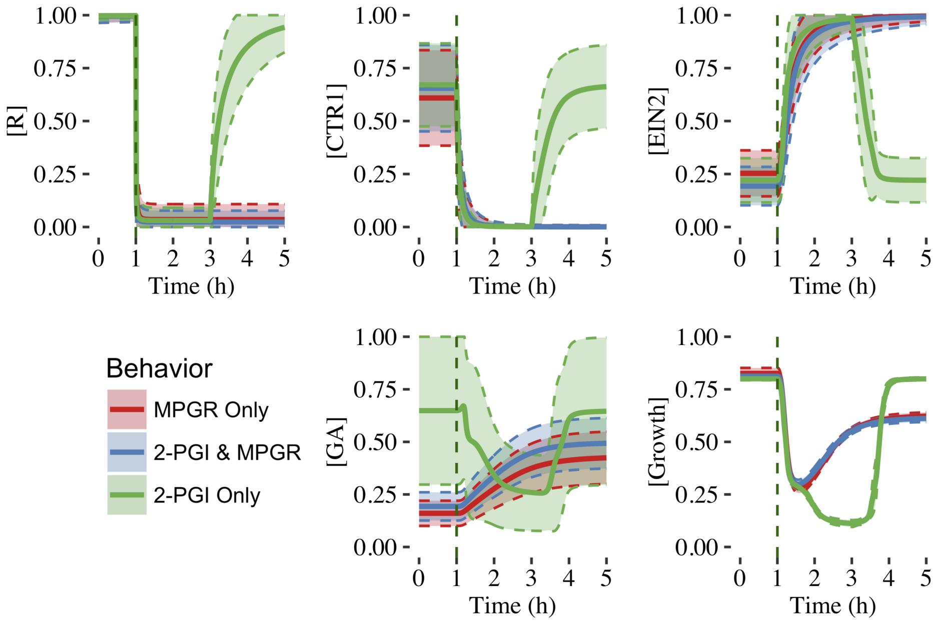

Figure S4. Time evolution of CFF/NFB network components (*ein3;eil1* mutant conditions). The mean response $\pm$ 1 standard deviation for all components from evolved CFF/NFB parameter sets passing 2-PGI and/or MPGR screening under *ein3;eil1* mutant saturating ethylene conditions.

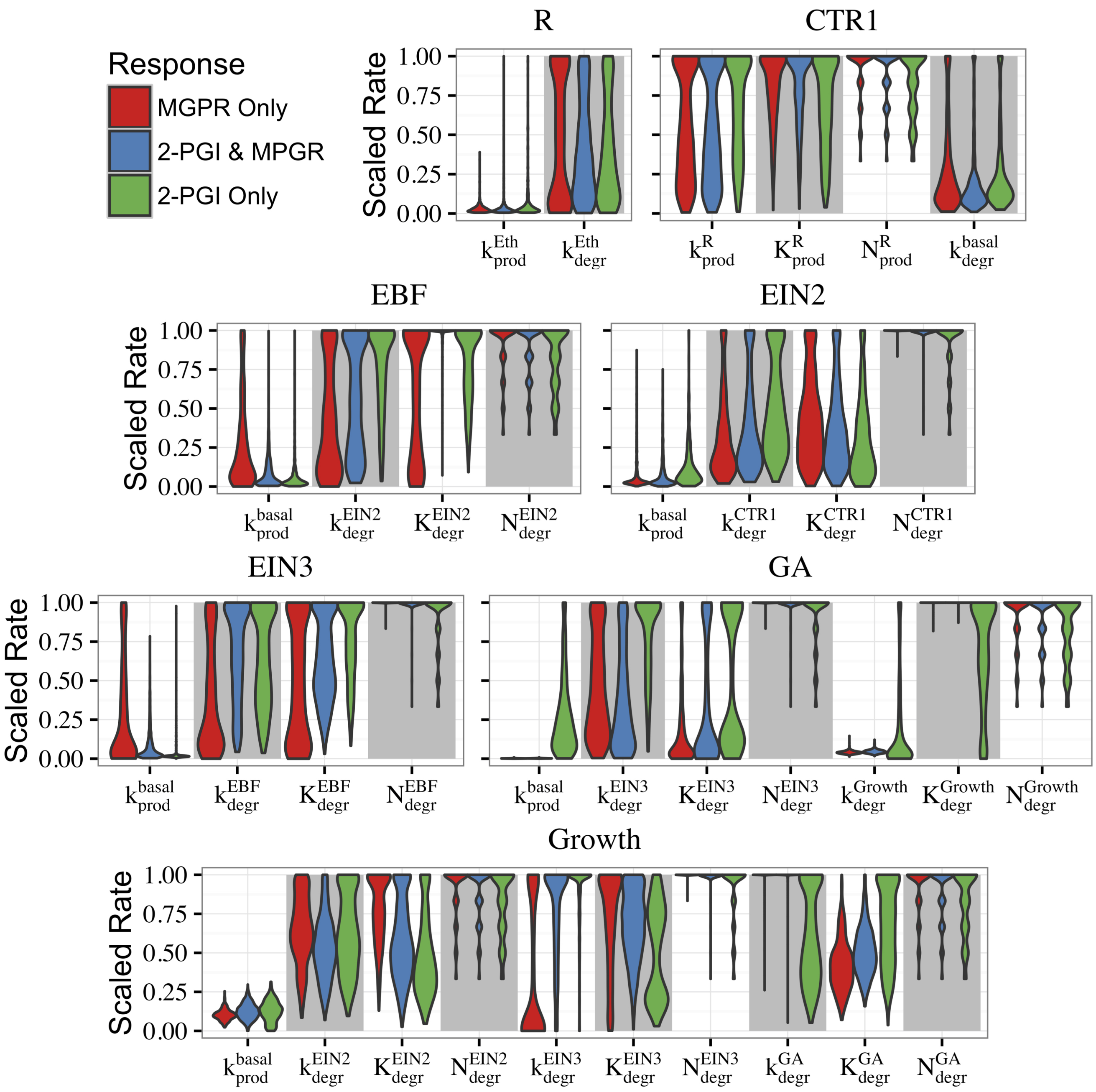

Figure S5. Complete parameter distributions for the CFF/NFB network. Parameter distributions of all CFF/NFB network parameters from evolved sets passing 2-PGI and/or MPGR screening. The width of each distribution is normalized such that the maximum width is equal across all parameters. All rates are unit normalized. Hill coefficients (N) are restricted to integer values.

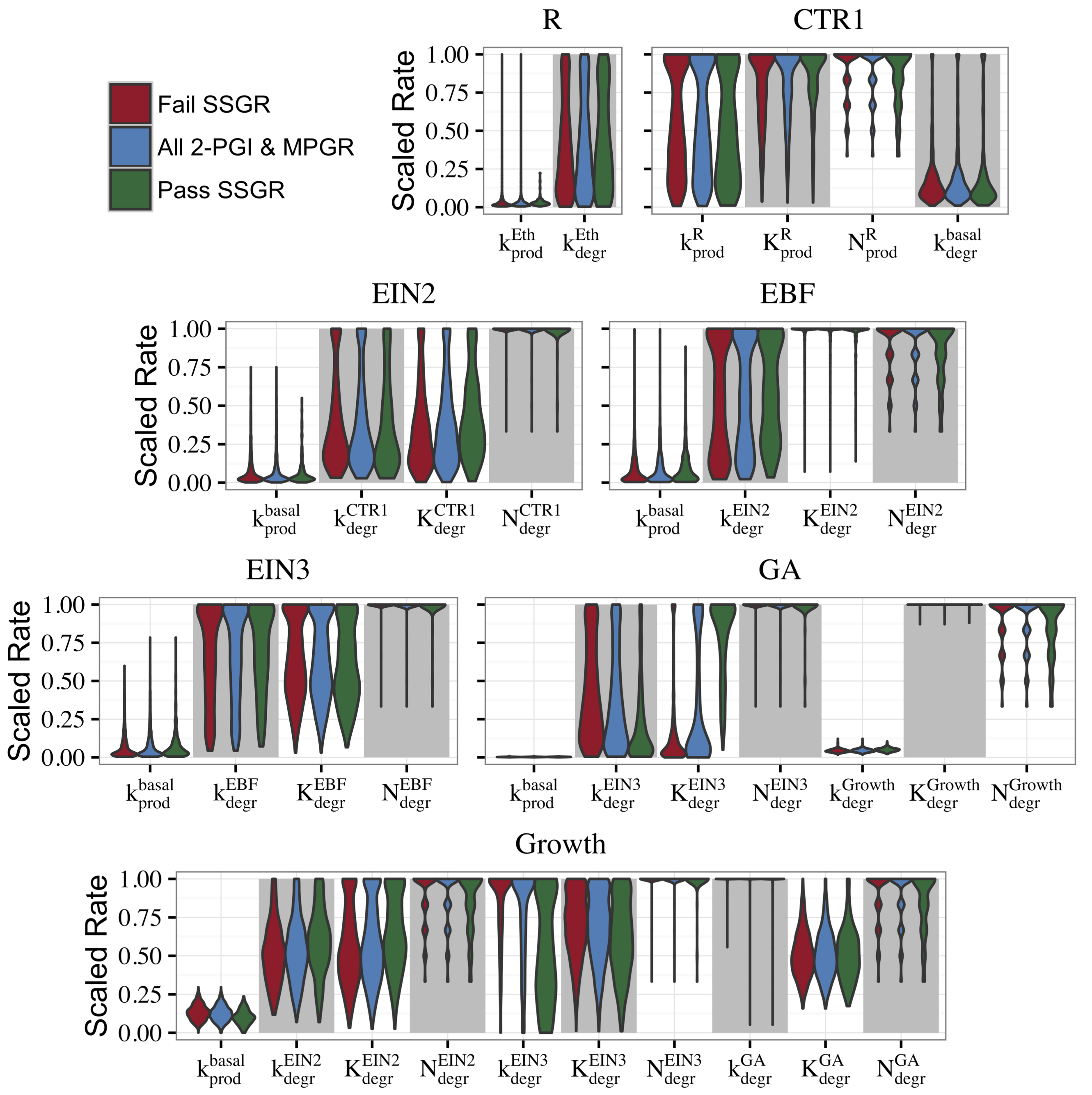

Figure S6. Parameter distributions for the CFF/NFB network based on SSGR screening. Comparison of the parameter distributions of all CFF/NFB network parameters for three cases: all sets passing both 2-PGI and MPGR screenings and the subset of these passing and failing SSGR screening. The width of each distribution is normalized such that the maximum width is equal across all parameters. All rates are unit normalized. Hill coefficients (N) are restricted to integer values.

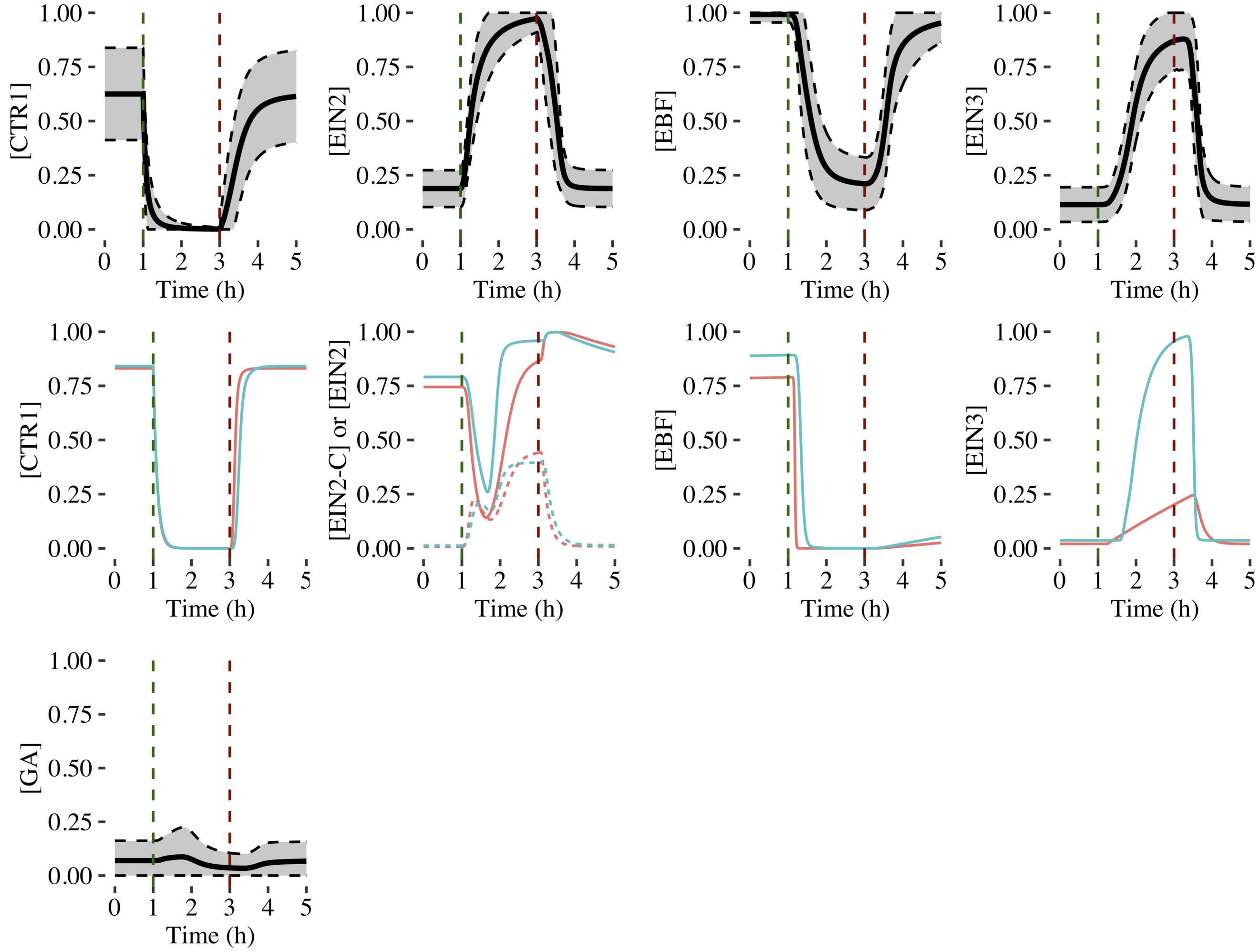

Figure S7. Time evolution of CFF/NFB and PFB network components (wildtype conditions). Comparison of the time evolution of components common to both the CFF/NFB (row 1) and PFB (row 2) networks. Results are from cases that exhibit 2-PGI, MPGR, and SSGR behavior. Row 1 shows the mean ± 1 standard deviation of responses from the CFF/NFB network. Row 2 shows the time evolution of components from the two evolved sets passing all screening from the PFB network. EIN2-C acts as the component inhibiting growth in the PFB network. This role is served by EIN2 in the CFF/NFB network. Comparing these components is illuminating for understanding the differences in the two networks. We plot together both EIN2-C (dashed line) and EIN2 (solid line) for the PFB network. Due to the critical nature of GA of the CFF/NFB network, we also plot its time evolution on the third row.
